# Role of IRE1α in podocyte proteostasis and mitochondrial health

**DOI:** 10.1038/s41420-020-00361-4

**Published:** 2020-11-19

**Authors:** José R. Navarro-Betancourt, Joan Papillon, Julie Guillemette, Takao Iwawaki, Chen-Fang Chung, Andrey V. Cybulsky

**Affiliations:** 1 Department of Medicine, McGill University Health Centre Research Institute, McGill University, Montreal, QC Canada; 2 Department of Life Science, Kanazawa Medical University, Uchinada, Japan

**Keywords:** Kidney, Glomerular diseases

## Abstract

Glomerular epithelial cell (GEC)/podocyte proteostasis is dysregulated in glomerular diseases. The unfolded protein response (UPR) is an adaptive pathway in the endoplasmic reticulum (ER) that upregulates proteostasis resources. This study characterizes mechanisms by which inositol requiring enzyme-1α (IRE1α), a UPR transducer, regulates proteostasis in GECs. Mice with podocyte-specific deletion of IRE1α (IRE1α KO) were produced and nephrosis was induced with adriamycin. Compared with control, IRE1α KO mice had greater albuminuria. Adriamycin increased glomerular ER chaperones in control mice, but this upregulation was impaired in IRE1α KO mice. Likewise, autophagy was blunted in adriamycin-treated IRE1α KO animals, evidenced by reduced LC3-II and increased p62. Mitochondrial ultrastructure was markedly disrupted in podocytes of adriamycin-treated IRE1α KO mice. To pursue mechanistic studies, GECs were cultured from glomeruli of IRE1α flox/flox mice and IRE1α was deleted by Cre–lox recombination. In GECs incubated with tunicamycin, deletion of IRE1α attenuated upregulation of ER chaperones, LC3 lipidation, and LC3 transcription, compared with control GECs. Deletion of IRE1α decreased maximal and ATP-linked oxygen consumption, as well as mitochondrial membrane potential. In summary, stress-induced chaperone production, autophagy, and mitochondrial health are compromised by deletion of IRE1α. The IRE1α pathway is cytoprotective in glomerular disease associated with podocyte injury and ER stress.

## Introduction

Secreted and membrane proteins are translocated into the endoplasmic reticulum (ER), where they are covalently modified to attain a correctly folded conformation by folding enzymes and chaperones, prior to transport to the Golgi and secretory pathway^1^. To maintain protein homeostasis (“proteostasis”) during cellular stress, the ER orchestrates the unfolded protein response (UPR), an adaptive signaling pathway activated by accumulation of misfolded proteins in the ER (i.e. “ER stress”)^2^. The UPR results in upregulation of ER chaperones, translational attenuation, and clearance of misfolded proteins^1^. Inositol requiring enzyme-1α (IRE1α), the most evolutionarily conserved UPR transducer, is an ER transmembrane kinase and endoribonuclease^3^. Upon accumulation of misfolded proteins, IRE1α homomultimerizes and transautophosphorylates to activate its endoribonuclease activity that removes an intron in XBP1 mRNA. Spliced XBP1 (XBP1s) mRNA encodes a transcription factor that translocates into the nucleus to activate genes that encode chaperones, autophagy mediators, and induce metabolic adaptations^1^. IRE1α signaling is essentially adaptive, but under sustained ER stress it may become cytotoxic/apoptotic^2^.

ER stress has been linked with macroautophagy (hereafter autophagy), a process that delivers long-lived proteins and organelles to lysosomes through sequestration of a cytoplasmic fraction within a membrane^4,5^. IRE1α can potentially promote autophagy through XBP1s-mediated transcription of autophagy effectors^6,7^, and phosphorylation/activation of c-Jun N-terminal kinase (JNK), which subsequently increases free Beclin-1 and activates phosphatidylinositol 3-kinase (PI3K)^4^. Mechanisms of ER stress-induced autophagy require further characterization.

Recent studies suggest that the ER communicates with other cell organelles. For example, the ER interacts with mitochondria, organelles that are key in maintaining cell energy homeostasis by synthesis of ATP^8^. The UPR and mitochondrial function are interrelated^9,10^. ER-mitochondrial contact sites, known as mitochondria-associated membranes (MAMs)^11^, are dynamic and can regulate mitochondrial metabolism, apoptosis, and autophagy^8^. IRE1α is enriched at MAMs^12^; however, its functional implications for ER-mitochondrial crosstalk are yet to be determined.

Glomerular epithelial cells (GECs)/podocytes are vital in maintaining glomerular capillary wall permselectivity^13^. Intact ER function is important for proteostasis in podocytes^14^, including production of components of the slit-diaphragm, adhesion complexes, and glomerular basement membrane (GBM)^13^. Protein misfolding in the ER (ER stress) contributes to the pathogenesis of human glomerular diseases^15^, in particular membranous nephropathy^16^, focal segmental glomerulosclerosis (FSGS)^17^, and diabetic nephropathy^18^. In podocytes, deletion of key autophagy genes leads to injury, implying that autophagy is important for proteostasis^19,20^. Autophagy deficiency induces ER dysfunction^20^, and autophagy is recognized as an adaptive mechanism during the UPR^21^. Nonetheless, how the UPR drives autophagy in podocytes is not fully understood.

In mice, podocyte-specific deletion of IRE1α leads to age-related podocyte injury and albuminuria, associated with impaired autophagy^22^. Whether IRE1α and ER stress play a pathogenic role in chronic glomerular diseases associated with podocyte injury warrants investigation. In the present study, we characterize the mechanism by which IRE1α mediates podocyte proteostasis in adriamycin nephrosis and demonstrate a novel role of IRE1α in maintaining mitochondrial health.

## Results

### IRE1α signaling is adaptive in adriamycin nephrosis

**Fig. 1:**
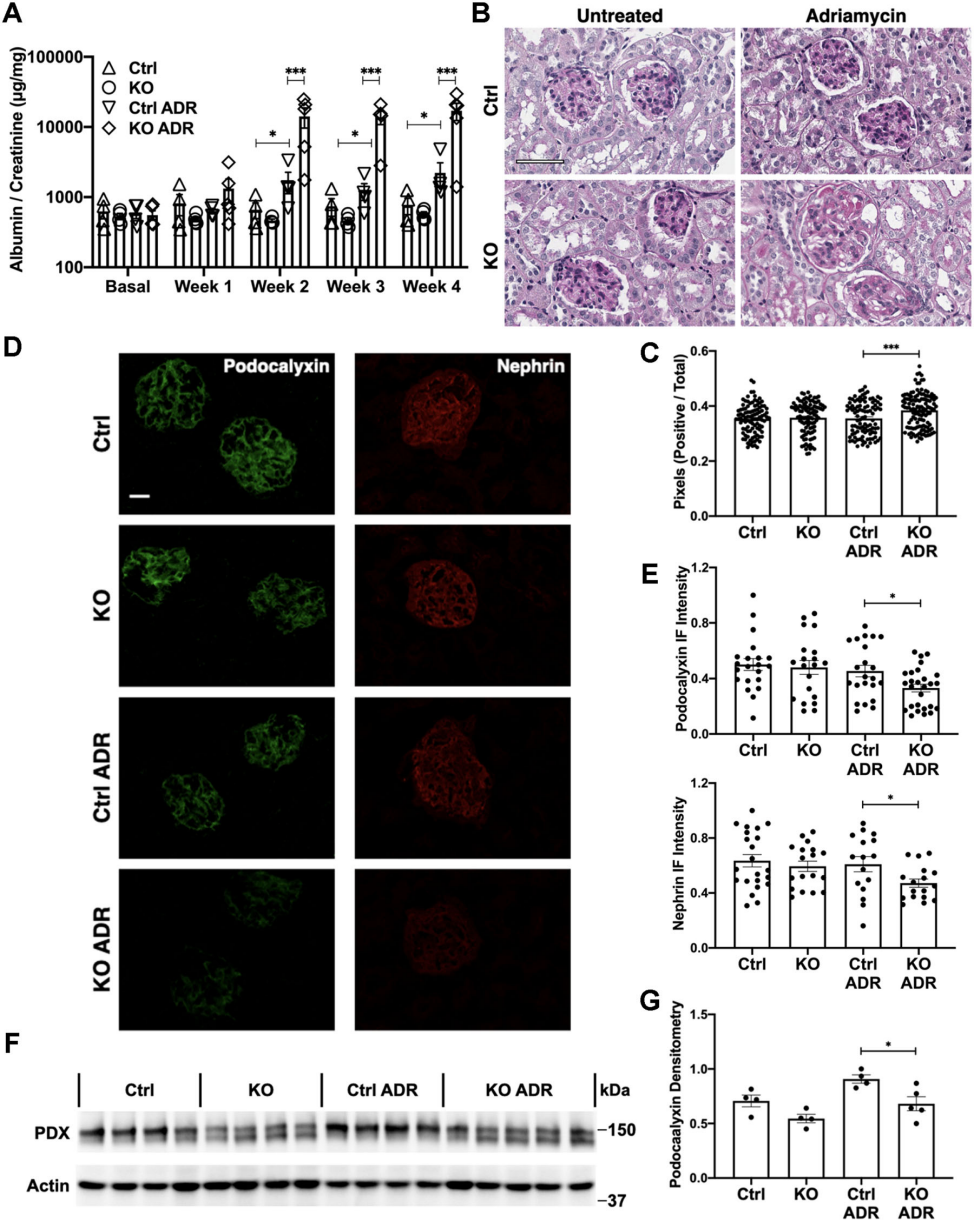
Podocyte-specific deletion of IRE1α exacerbates podocyte injury in adriamycin (ADR) nephrosis. **A** Albuminuria was exacerbated in ADR-treated IRE1α KO mice, compared with ADR-treated control (Ctrl) littermates. There are 4 mice per group, except 5 mice in the KO ADR group. **B** Representative images of glomerular histology (PAS stain). Bar = 60 μm. **C** Glomerular matrix expansion, evaluated with a pixel-counting algorithm, was greater in IRE1α KO mice than in control animals after ADR injection. 25 glomeruli in 4–5 mice per group were analyzed. **D** Kidney sections were stained for podocalyxin and nephrin. Bar = 20 μm. **E** Compared with the ADR-treated control group, podocalyxin and nephrin fluorescence intensity was lower in treated IRE1α KO mice. 16–27 glomeruli in 4–5 mice per group were analyzed. **F**, **G** Podocalyxin (PDX) runs as a doublet or a smear due to various post-translational modifications; the entire podocalyxin signal was quantified. PDX expression was reduced in glomerular lysates of IRE1α KO mice (immunoblot). *P ≤ 0.05, **P ≤ 0.01, ***P ≤ 0.001.

We addressed the functional role of IRE1α in podocyte injury in mice by inducing adriamycin nephrosis, a model of chronic proteinuric glomerular disease resembling human FSGS. Baseline urine albumin/creatinine ratio was similar in control and IRE1α KO mice (Fig. 1A). Compared with untreated mice, albuminuria in adriamycin-treated control mice increased significantly over 4 weeks. Moreover, adriamycin-induced albuminuria was substantially greater in IRE1α KO mice (Fig. 1A).

Glomerular histology, including morphometric quantification, revealed that adriamycin-treated KO mice displayed increases in glomerular extracellular matrix compared with injected controls (Fig. 1B, C, Supplementary Fig. 1C). Immunofluorescence microscopy showed reduced glomerular expression of the podocyte differentiation markers podocalyxin and nephrin in adriamycin-treated IRE1α KO mice, compared with treated controls (Fig. 1D, E). By analogy, immunoblotting showed reduced expression of podocalyxin in glomerular lysates of adriamycin-treated IRE1α KO mice (Fig. 1F, G). However, differences in nephrin were not significant (Supplementary Fig. 1D, E).

**Fig. 2:**
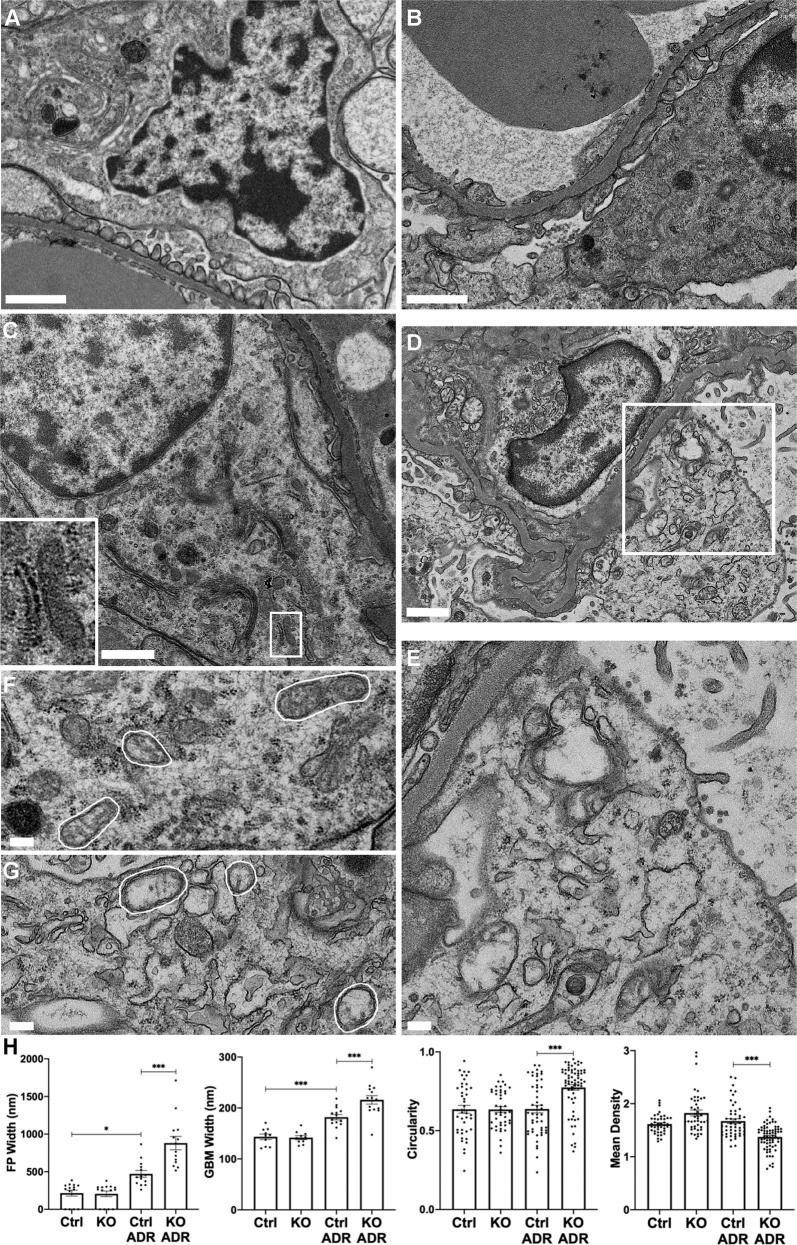
Deletion of IRE1α in podocytes exacerbates podocyte ultrastructural injury in adriamycin (ADR) nephrosis. **A**–**G** Representative electron micrographs of untreated or ADR-treated control and IRE1α KO mice. Podocyte foot process architecture and organelles are normal in untreated control (Ctrl; **A**) and KO mice **B**. There is some foot process and GBM widening in ADR-treated Ctrl mice, although podocyte organelles appear normal **C**. A normal MAM is shown in **C** (inset). ADR-treated KO mice show focal foot process effacement, microvillous transformation of podocyte plasma membranes, widening of the GBM, as well as swelling of the ER and damage to mitochondria in podocytes **D**, **E**. Mitochondrial damage was quantified by measuring circularity and matrix density; representative mitochondria in ADR-treated Ctrl **F** and KO mice **G** are outlined with white lines. **H** Quantification of foot process (FP) and GBM width, as well as mitochondrial circularity and matrix density. Scale bars: **A**–**D** = 1 μm, **E**–**G** = 200 nm. *P ≤ 0.05, ***P ≤ 0.001. 2–3 glomeruli, 10–14 capillary loops (FP and GBM width), and 43–68 mitochondria per mouse were examined in 3 mice per group.

By electron microscopy, untreated control and KO mice showed normal podocyte foot processes and organelles (Fig. 2A, B). Foot processes demonstrated widening in adriamycin-treated control mice, although podocyte organelles appeared normal (Fig. 2C, H). Adriamycin-treated KO mice showed focal foot process effacement and overall widening, as well as microvillous transformation and vesiculation of podocyte plasma membranes (Fig. 2D, E, H). Compared with untreated control, adriamycin-treated control mice demonstrated GBM widening, and there was further widening in treated KO mice (Fig. 2H). The latter is in keeping with expansion of glomerular extracellular matrix (Fig. 1B, C). Ultrastructural features of podocyte apoptosis were not evident in any glomeruli. Compared with treated controls, remarkable injury of podocyte organelles was evident in adriamycin-treated IRE1α KO mice, including ER dilatation and fragmentation of Golgi cisternae (Fig. 2D, E). Interestingly, mitochondrial damage was prominent in these animals; ultrastructural changes included disruption of cristae, increased circularity (loss of elongation), and loss of matrix density (Fig. 2D–H). These results suggest that IRE1α is important in preserving mitochondrial integrity.

### IRE1α mediates ER chaperone synthesis and autophagy in vivo

**Fig. 3:**
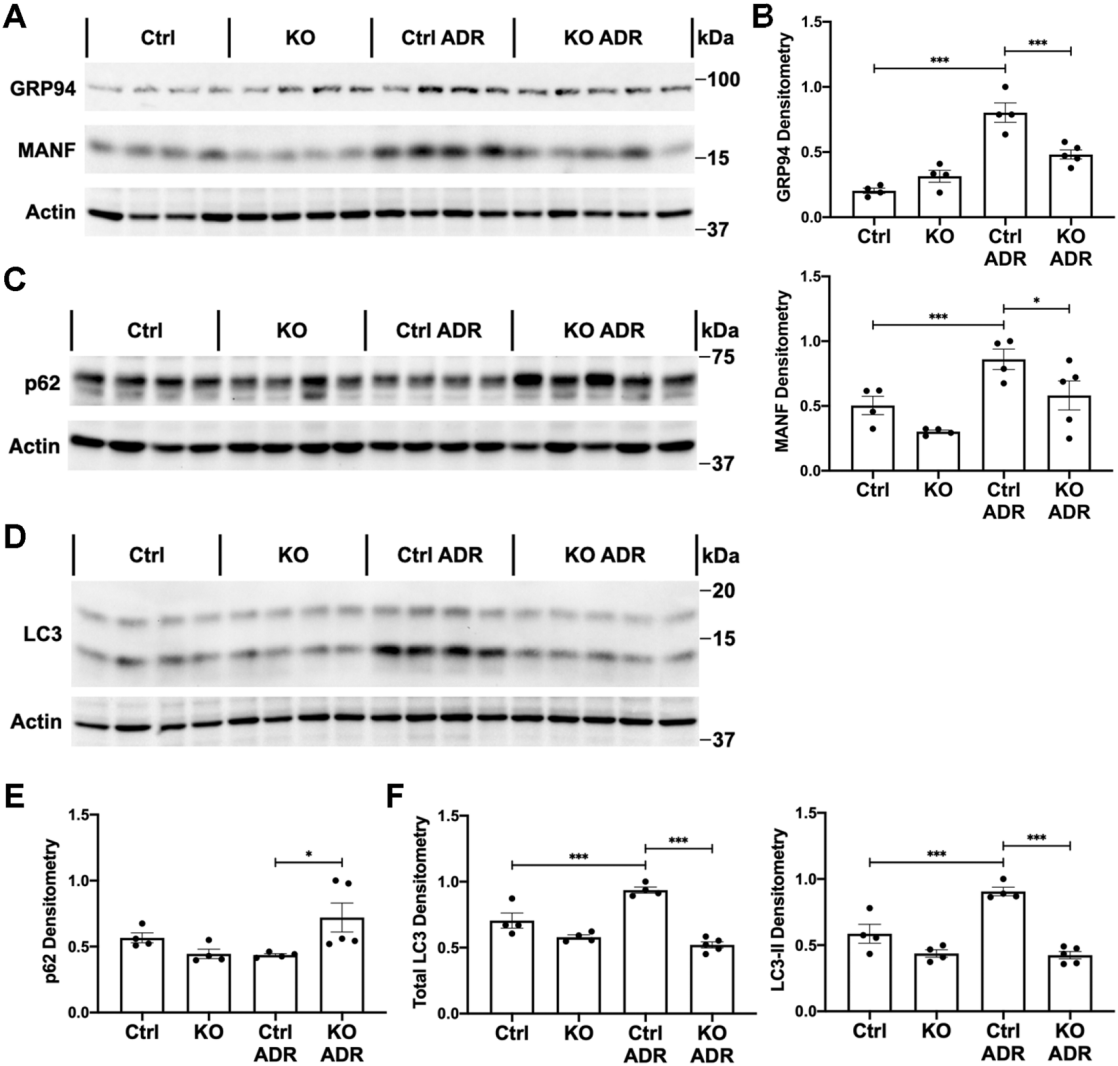
Upregulation of glomerular autophagy markers and ER chaperones in adriamycin (ADR) nephrosis is attenuated in IRE1α KO mice. **A**, **B** By immunoblotting, expression of the chaperones GRP94 and MANF was lower in IRE1α KO mice treated with ADR, compared with treated control (Ctrl). **C**, **E** Accumulation of the autophagy substrate p62 in glomeruli of ADR-treated IRE1α KO mice indicates reduced autophagic flux. **D**, **F** Total and lipidated LC3 (LC3-II) increased in adriamycin nephrosis; this upregulation was impaired in IRE1α KO mice, indicating reduced autophagosome biogenesis. *P ≤ 0.05, ***P ≤ 0.001. There are 4–5 mice per group.

In these experiments, we addressed potential mechanisms by which IRE1α protected podocytes from injury. There were no significant differences in basal levels of glomerular ER chaperones GRP94 and MANF in IRE1α KO mice, compared with control (Fig. 3A, B). In control mice, adriamycin-stimulated GRP94 and MANF, indicating induction of the UPR; however, upregulation of GRP94 and MANF was attenuated in treated IRE1α KO mice (Fig. 3A, B). Thus, IRE1α activity is required for effective production of these chaperones.

Next, we monitored formation of autophagosomes by lipidation of LC3 (i.e. LC3-II), and autophagic flux by changes in the autophagy substrate p62^5^. These parameters did not differ significantly in untreated mice (Fig. 3C–F). In response to adriamycin, control mice displayed LC3-II accumulation, in keeping with enhanced autophagosome biogenesis. Autophagy was blunted in adriamycin-treated IRE1α KO mice, evidenced by reduced LC3-II and increased p62 compared with treated controls (Fig. 3C–F). The latter is consistent with deficient autophagic flux. Of note, total LC3 expression was greater in treated control mice, compared with the treated KO (Fig. 3D, F).

### IRE1α mediates ER chaperone synthesis and autophagy in GECs

**Fig. 4:**
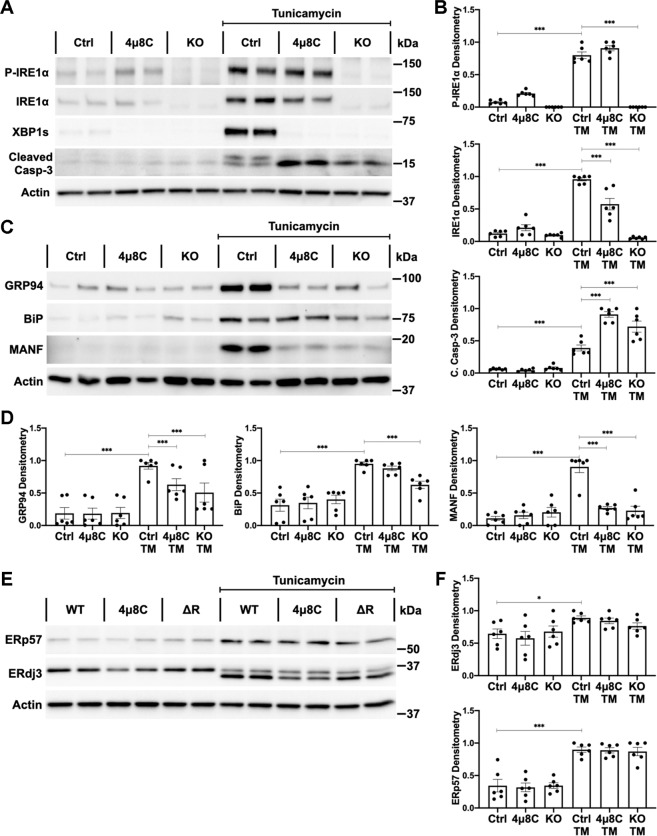
IRE1α signaling is adaptive in cultured GECs subjected to proteotoxic stress. **A**, **B** Incubation with TM for 24 h stimulated the upregulation of total IRE1α, robust XBP1 splicing, and mild apoptosis (caspase-3 cleavage) in control (Ctrl) GECs. Quantification of IRE1α phosphorylation was normalized to the actin signal. IRE1α phosphorylation was absent in IRE1α KO GECs but was not affected by 4μ8C, compared to control GECs. IRE1α KO and 4μ8C-treated GECs show undetectable XBP1 splicing and greater apoptosis (immunoblots). **C**–**F** TM increased the expression of ER chaperones in control GECs. **C**, **D** Upregulation of GRP94, BiP and MANF was impaired in IRE1α KO GECs, and these results were generally recapitulated by 4μ8C. **E**, **F** The chaperone ERdj3 is a glycosylated protein. TM inhibits glycosylation and induces an underglycosylated band of ~34 kDa (both bands were used for quantification). Upregulation of ERp57 and ERdj3 was not affected by IRE1α inhibition. *P ≤ 0.05, **P ≤ 0.01, ***P ≤ 0.001. Three experiments performed in duplicate.

To further delineate how IRE1α regulates podocyte proteostasis, we generated cultured mouse GECs with deletion of IRE1α. Under basal conditions, IRE1α protein and phosphorylation were present in control GECs and control GECs treated with the IRE1α ribonuclease inhibitor 4μ8C, but not in IRE1α KO GECs (Fig. 4A, B). We induced protein misfolding and ER stress by incubation with TM. IRE1α autophosphorylation may precede activation of its ribonuclease^23^. TM induced IRE1α phosphorylation in control, but not IRE1α KO GECs, indicating loss of kinase activity in KO cells (Fig. 4A, B). 4μ8C did not affect TM-induced IRE1α phosphorylation, implying an uncoupling of kinase and ribonuclease signaling^24^. Interestingly, IRE1α itself behaves as an ER stress response protein, since TM increased IRE1α expression in control GECs. Compared with TM-treated control, stress-induced IRE1α upregulation was blunted by 4μ8C and was absent in IRE1α KO GECs (Fig. 4A, B).

XBP1s was translated in TM-treated control GECs, but was undetectable in TM-treated IRE1α KO and TM + 4μ8C-treated control cells, confirming absence of ribonuclease activity (Fig. 4A). The absence of XBP1 splicing in IRE1α KO and 4μ8C-treated control GECs was associated with enhanced ER stress-induced apoptosis, evidenced by an elevation of cleaved caspase-3 (Fig. 4A, B). Thus, IRE1α signaling is cytoprotective/adaptive.

Basal levels of ER chaperones did not differ among control GECs, control GECs treated with 4μ8C and KO GECs. In control GECs, expression of BiP, GRP94, ERp57, ERdj3, and MANF increased after 24 h of TM exposure (Fig. 4C–F). MANF and ERdj3 are ER chaperones that may be secreted from cells^25^. Accordingly, MANF and ERdj3 were also elevated in TM-conditioned media (Supplementary Fig. 3A, B). After 24 h of TM incubation, deletion of IRE1α significantly attenuated the upregulation of BiP, GRP94, and MANF (Fig. 4C, D). Chemical inhibition of IRE1α with 4μ8C impaired the expression of GRP94 and MANF, but not of BiP (Fig. 4C, D). Neither IRE1α deletion nor 4μ8C treatment affected the upregulation of ERp57 and ERdj3, compared with stressed control GECs (Fig. 4E, F). Moreover, extracellular MANF was decreased by IRE1α inhibition, while ERdj3 was unaffected (Supplementary Fig. 3A, B). Thus, IRE1α is essential for effective stimulation of certain ER chaperones, while other chaperones may be activated predominantly via the ATF6 pathway^26^.

Upregulation of chaperones in response to ER stress is reported to occur in a time-dependent manner^27^. At an earlier time point (8 h of TM treatment), there were increases in BiP, GRP94 and MANF in control GECs, but changes were less robust compared with 24 h incubations. IRE1α deletion and 4μ8C treatment impaired the upregulation of GRP94, BiP, and MANF, compared with treated control cells (Supplementary Fig. 3C, D). Interestingly, the impairment of MANF production caused by IRE1α inhibition was more marked than the defects in GRP94 or BiP upregulation (Fig. 4C, D, Supplementary Fig. 3C, D).

**Fig. 5:**
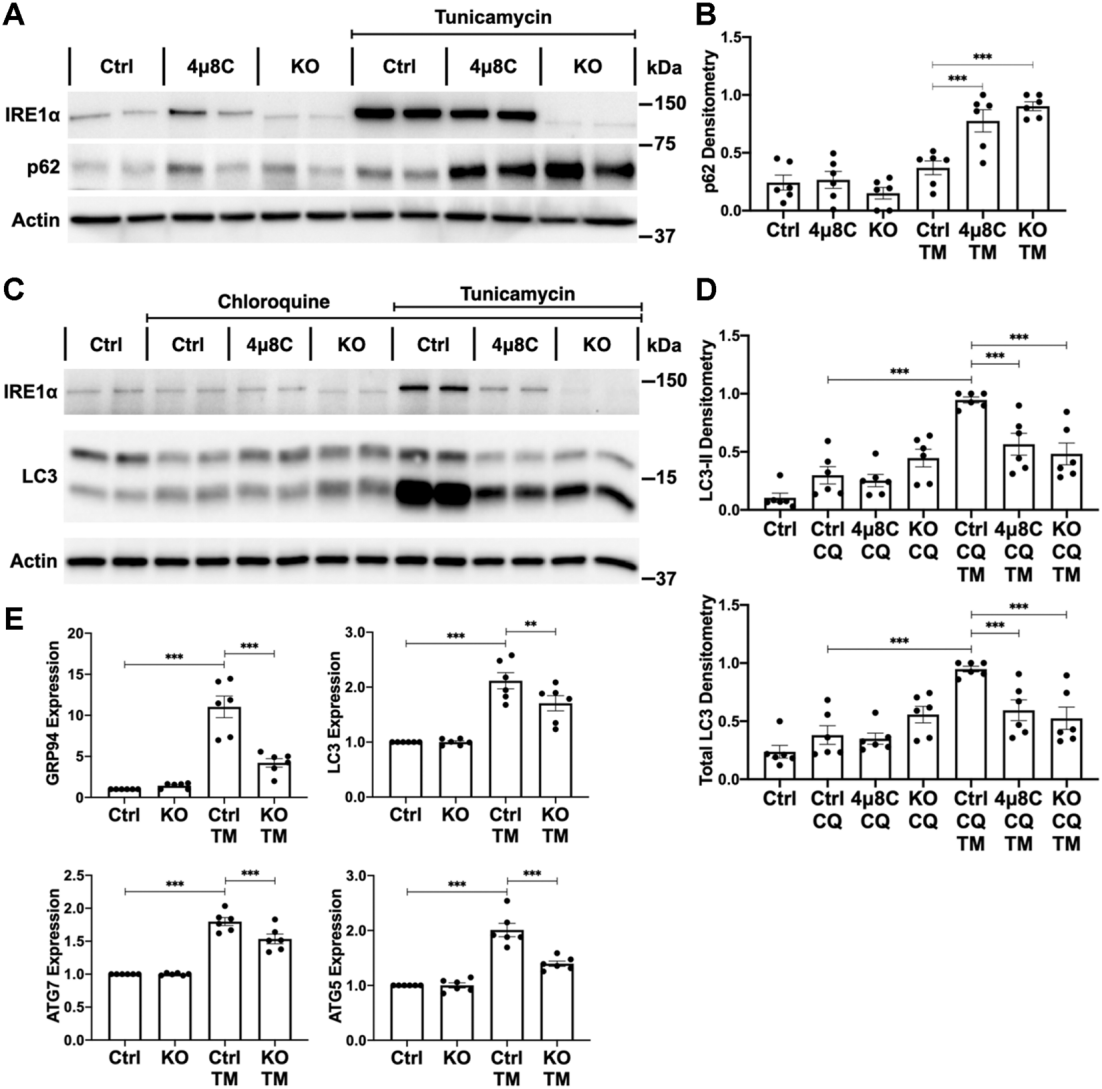
IRE1α stimulates autophagy transcriptionally in GECs. **A**, **B** TM treatment (24 h) induced significant accumulation of the autophagy substrate p62 in GECs with genetic deletion and chemical inhibition (4μ8C) of IRE1α (immunoblot). **C**, **D** In control (Ctrl) GECs, total and lipidated LC3 (LC3-II) increased markedly with chloroquine (CQ) and TM co-incubation; IRE1α deletion and 4μ8C decreased the upregulation of total LC3 and LC3-II. (CQ blocks the fusion of autophagosomes with lysosomes and prevents autolysosomal protein degradation, allowing comparison of the rates of autophagosome formation.) **E** Compared with TM-treated control GECs, TM-induced transcription of GRP94, LC3 (MAP1LC3B), ATG5, and ATG7 mRNAs was impaired in IRE1α KO GECs (qPCR). *P ≤ 0.05, **P ≤ 0.01, ***P ≤ 0.001. Three experiments performed in duplicate.

Autophagic flux was monitored by changes in p62. In control and KO GECs, basal levels of p62 were similar. Compared with TM-treated control GECs, IRE1α inhibition and deletion resulted in accumulation of p62 (24 h incubation with TM), which denotes deficient autophagic flux (Fig. 5A, B). The rate of autophagosome formation was measured by lipidation of LC3 in the presence of chloroquine. Basal autophagosome formation did not change significantly at 8 or 24 h (Supplementary Fig. 4A, B, and Fig. 5C, D). TM-induced autophagy was unchanged at 8 h (Supplementary Fig. 4A, B). After 24 h, TM significantly increased LC3-II and total LC3 in control GECs, but 4μ8C-treated control and IRE1α KO GECs showed impaired upregulation of LC3-II and total LC3 (Fig. 5C, D). This indicates reduced autophagosome biogenesis and implies defective LC3 transcription, respectively.

### IRE1α regulates autophagy transcriptionally

Since the effects of IRE1α deletion were generally replicated by inhibition of ribonuclease activity, and the induction of autophagy occurred 24 h after induction of ER stress, we hypothesized that IRE1α may, at least in part, promote autophagy through transcriptional upregulation of autophagy mediators. First, we identified autophagy-related genes with potential XBP1s-binding sites^28^ (Supplementary Fig. 5A). Then, we measured the candidate mRNAs using qPCR. In control GECs, TM increased mRNAs of MAP1LC3B (LC3), ATG5, and ATG7 ~2-fold, compared with unstimulated (Fig. 5E). These increases were attenuated significantly in stimulated IRE1α KO GECs. Other XBP1 target mRNAs, including BECN1, ATG12, and PI3K catalytic subunit type 3 increased comparably in control and IRE1α KO GECs (Supplementary Fig. 5). For comparison, TM increases GRP94 (1 XBP1-binding site) mRNA more than 10-fold in control GECs and very weakly in IRE1α KO GECs (Fig. 5E). Thus, transcription of certain autophagy genes is dependent on IRE1α-XBP1s, although the number of XBP1-binding sites in a gene correlates poorly with the level of transcription.

It should be noted that ER stress induced the upregulation of total LC3 protein, and this was attenuated by IRE1α deletion in vivo (Fig. 3D, F) and in culture (Fig. 5C, D), which is consistent with a defect in the transcription of MAP1LC3B. We did not, however, detect TM-stimulated increases in Atg5 or Atg7 at the protein level (data not shown).

Phosphorylation of IRE1α has been associated with induction of autophagy via JNK activation, dissociation of Beclin-1 from Bcl-2 and assembly of the autophagy initiation complex^4,29^. Although IRE1α phosphorylation was impaired in IRE1α KO GECs (Fig. 4A, B), TM increased activation-specific phosphorylation of JNK comparably in control and IRE1α KO cells, while total Beclin-1 remained unchanged (Supplementary Fig. 6A–D). To monitor dissociation of Beclin-1 from Bcl-2, we coimmunoprecipitated endogenous Bcl-2 and Beclin-1 with an anti-Bcl-2 antibody^29^. TM treatment did not modify the amount of Beclin-1 that coimmunoprecipitated with Bcl-2, indicating no effect on Beclin-1 dissociation (Supplementary Fig. 6E, F). These results do not support the IRE1α-phospho-JNK-Bcl-2 axis as a stress-induced autophagy mechanism.

### IRE1α sustains the mitochondrial oxygen consumption rate

**Fig. 6:**
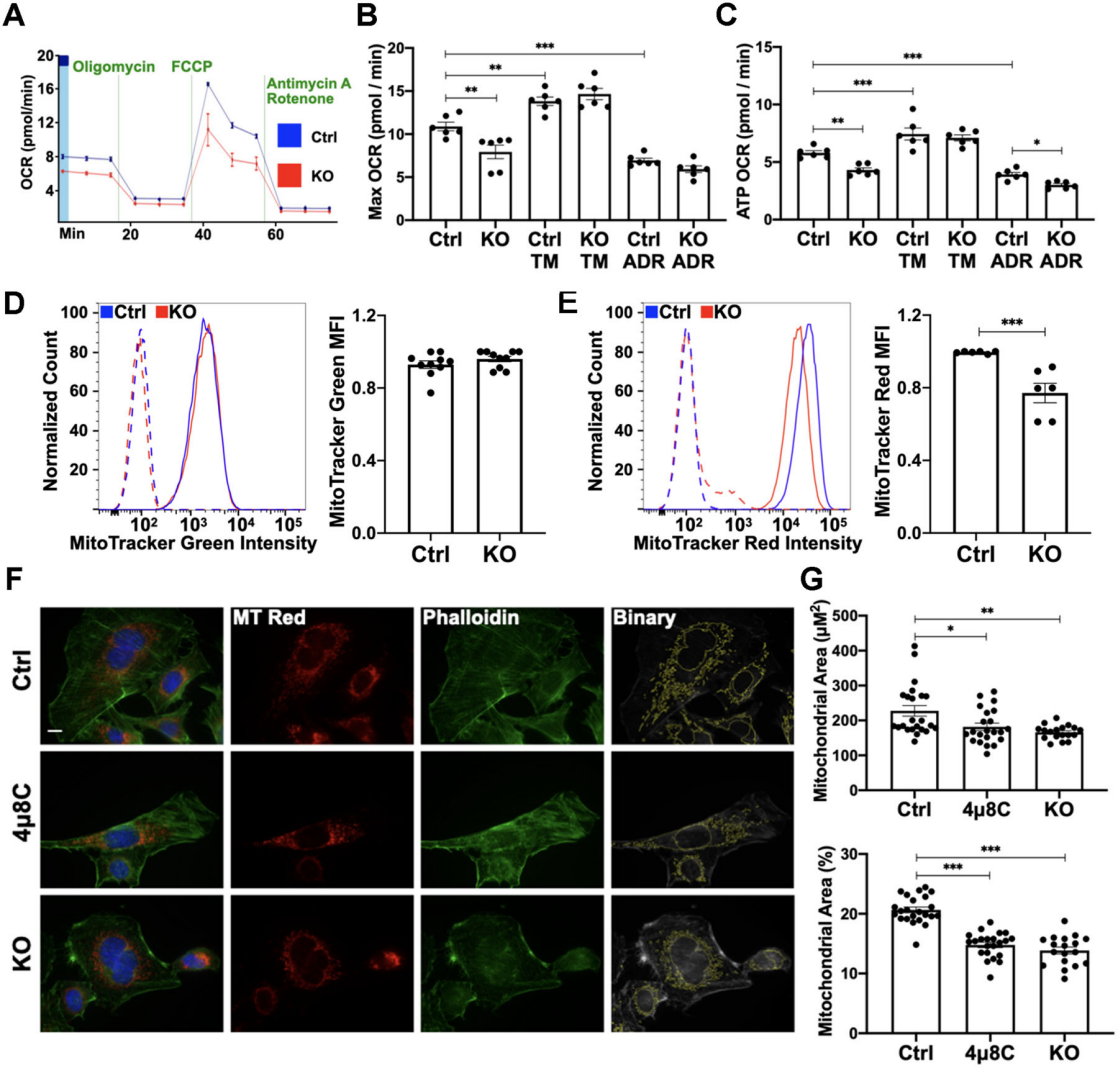
Deletion of IRE1α impairs mitochondrial metabolism in GECs. **A** OCR was measured with the Seahorse mitochondrial stress test. **B**, **C** Compared with control (Ctrl) cells, maximal and ATP-linked OCR were reduced in IRE1α KO GECs under resting conditions. TM treatment (24 h) increased maximal and ATP-linked OCR (respiration linked to ATP production), while adriamycin (ADR) decreased these values. ATP-driven respiration after ADR exposure was further reduced in IRE1α KO GECs. Experiment of six biological replicates, representative of three experiments. **D** MitoTracker Green fluorescence intensity was similar between control and IRE1α KO GECs. Five experiments performed in duplicate. **E** MitoTracker Red fluorescence intensity was significantly lower in IRE1α KO GECs. The profiles of unstained cells are shown with dashed lines. Three experiments performed in duplicate. **F** Representative images of GECs stained with MitoTracker Red and FITC-phalloidin. Mitochondria are circumscribed in the binary images. Scale bar = 10 μm. **G** Deletion or inhibition (4μ8C) of IRE1α decreased the area labeled with MitoTracker Red (total mitochondrial area and mitochondrial area as % of total cell area). 18–23 frames were measured per group. *P ≤ 0.05, **P ≤ 0.01, ***P ≤ 0.001.

The disruption of mitochondrial ultrastructure in adriamycin-treated IRE1α KO mice was striking and prompted us to examine mitochondrial function in GECs. Compared with control, IRE1α KO GECs showed a decreased maximal OCR, indicating mitochondrial dysfunction and reduced capacity for ATP production (Fig. 6A, B, Supplementary Fig. 7A). Maximal OCR increased after exposure to TM (24 h); however, no differences were detected between control and KO GECs. Inhibition of cytochrome c oxidase with adriamycin reduced maximal OCR in control GECs. There was no further reduction in KO cells, as the OCR was already markedly reduced (Fig. 6B, Supplementary Fig. 7A).

Under resting conditions, mitochondrial metabolism was reduced in IRE1α KO GECs, evidenced by a lower ATP-linked OCR compared with control (Fig. 6C, Supplementary Figure 7B). TM increased ATP-linked respiration in both cell lines and adriamycin exposure decreased ATP-linked OCR; the decrease was greater in IRE1α KO GECs (Fig. 6C), consistent with aggravated mitochondrial injury in the absence of IRE1α.

To assess whether reduction of OCR in IRE1α KO GECs was the consequence of a lower mitochondrial mass or a functional defect, mitochondria were labeled with MitoTracker Green FM. Separately, MitoTracker Red CMXRos, which accumulates inside the mitochondrial matrix in a membrane potential-dependent fashion, was used to evaluate mitochondrial activity^30^. Flow cytometry revealed no differences in forward light scatter (Supplementary Fig. 7C) and MitoTracker Green fluorescence intensity between control and IRE1α KO GECs (Fig. 6D), indicating similar cell size and total mitochondrial mass. Compared with control, MitoTracker Red fluorescence was decreased significantly in IRE1α KO GECs (Fig. 6E), consistent with reduced mitochondrial function.

In a complementary approach, mitochondria were labeled with MitoTracker Red CMXRos and the actin cytoskeleton was stained with FITC-phalloidin (to monitor cell area). There were no significant differences in cell area between control, 4μ8C-treated control, and IRE1α KO GECs (Supplementary Fig. 7D), but inhibition or deletion of IRE1α decreased the area labeled with MitoTracker Red (Fig. 6F, G). Levels of PGC1α (a master regulator of mitochondrial biogenesis)^31^ were similar among control, 4μ8C-treated control, and IRE1α KO GECs under basal conditions and after TM stimulation, in keeping with the comparable total mitochondrial mass (Supplementary Fig. 7E, F). Together, these results indicate that in IRE1α KO GECs, mitochondrial respiration is impaired due to an intrinsic mitochondrial defect and without a compensatory increase in mitochondrial biogenesis.

### Human FSGS is associated with activation of the UPR

To determine if results in adriamycin nephrosis pertain to human disease, we interrogated the Nephroseq database, which contains glomerular gene expression data from human kidney biopsies. Among 176 ER genes, 45 were increased significantly in FSGS compared with healthy controls (Supplementary Table 2). Among these 45 genes, 17 are inducible by XBP1s^26^, implying activation of the IRE1α/XBP1 axis in human FSGS. Indeed, the top gene in FSGS is MANF, which is also induced in adriamycin nephrosis in an IRE1α-dependent manner (Fig. 3A, B). Gene ontology enrichment analysis using all genes upregulated in FSGS showed significant activation of pathways related to ER function, ER stress, and protein misfolding (Supplementary Table 3). Principal component analysis of changes in ER/UPR gene expression in FSGS indicates that FSGS patients are clearly distinguished from normal controls (Supplementary Fig. 8). Together these results substantiate that IRE1α and the UPR are active in human FSGS.

## Discussion

This study demonstrates a key role for IRE1α in protecting podocytes from injury in experimental FSGS. IRE1α integrates three homeostatic pathways, including an increase in the production of ER chaperones, autophagy, and mitochondrial bioenergetics. Thus, in adriamycin nephrosis, podocyte-specific IRE1α deletion exaggerated albuminuria and podocyte injury, impaired proteostasis, including production of ER chaperones and autophagy, and induced mitochondrial ultrastructural damage. Earlier, we demonstrated an important role for IRE1α in maintaining podocyte integrity as mice age^22^. In podocytes, proteins processed in the ER are critical to the maintenance of glomerular permselectivity^13^. Consequently, ER function requires robust adaptation to challenges imposed by physiologic demands and pathologic perturbations. Cytoprotective responses to stress include upregulation of ER chaperones that assist in the structural maturation of newly synthesized proteins and prevent aggregation^1^. Upregulation of major ER chaperones may require input from the three UPR branches^2^; nonetheless, certain UPR chaperones preferentially fall within the transcriptional scope of IRE1α–XBP1s, possibly in a cell type-dependent fashion. In podocytes, the IRE1α–XBP1s axis is indispensable for upregulation of MANF and plays a major role in upregulation of BiP and GRP94. ER chaperones most likely contribute to the cytoprotective effect of IRE1α in adriamycin nephrosis.

Autophagy is another adaptive proteostasis resource in podocytes, and indeed autophagy mitigates podocyte injury in adriamycin nephrosis^14,32^. It is reasonable to conclude that autophagy contributed to the cytoprotective effect of IRE1α in adriamycin nephrosis, as deletion of IRE1α impaired autophagosome formation and autophagic flux; mechanistically, we observed deficient upregulation of LC3 mRNA and total protein. Traditionally, stress-induced autophagy has focused on post-translational mechanisms^4^. Recently, transcriptional regulation of autophagy has received considerable interest, particularly when autophagy is sustained over a longer time frame and the pool of autophagy mediators needs to be replenished^33^. In GECs, IRE1α-XBP1s is essential for ER stress-induced transcription of LC3, ATG5, and ATG7. Proteins encoded by these three genes participate in autophagosome formation, which is believed to be the rate-limiting step in the autophagic process^5^. IRE1α ribonuclease inhibition generally replicated defects caused by IRE1α deletion and autophagosome biogenesis was relatively late after stress induction. Therefore, IRE1α reprograms the podocyte proteostasis network transcriptionally.

XBP1s can heterodimerize with distinct transcription factors^34^; consequently, its transcriptional targets may depend on the cell type^35^. In other cells, transcriptional targets of XBP1s include additional autophagy-related genes, e.g. BECN1^6^ and ATG3^7^. Moreover, IRE1α can splice transcripts with structure similar to the XBP1 cleavage site, including microRNAs, thereby fine-tuning autophagy^36^. Although IRE1α can also modulate autophagy post-translationally (via JNK, Beclin-1, and Bcl-2)^4^, our results did not implicate this axis in podocytes.

Recent research indicates that UPR signaling may extend beyond canonical protein folding and degradation^37^. Remarkably, deletion of IRE1α in podocytes led to a mitochondrial defect, revealing a novel role for IRE1α in organelle crosstalk. Communication between organelles is essential to maintain proteostasis^38^, however, the structural and functional interplay between the ER and the mitochondrial network is poorly understood. For the first time, we documented that a UPR transducer is involved in mitochondrial function in podocytes.

Deletion of IRE1α accentuated ultrastructural mitochondrial damage in adriamycin nephrosis. Adriamycin can injure glomerular cells through multiple mechanisms and induce local inflammation^39^. Adriamycin is reported to inhibit cytochrome c oxidase^40^, a component of the electron transport chain. Podocyte mitochondrial injury has been shown in other models of adriamycin-induced nephrotic syndrome^41,42^; however, exaggerated glomerular injury and albuminuria in adriamycin-treated IRE1α KO mice reveals an uncharacterized role of IRE1α in the maintenance of mitochondrial structure and bioenergetics.

In cultured GECs, maximal and ATP-coupled mitochondrial OCR were reduced by IRE1α deletion even under basal conditions, consistent with a report in another cell line^43^. The importance of resting mitochondrial metabolism for podocyte homeostasis remains to be determined^44^; nonetheless, in the context of disease, mitochondrial function appears to be essential for podocyte health^45,46^. Of note, after incubation with adriamycin, ATP-linked respiration was further reduced in IRE1α KO GECs, consistent with the prominent mitochondrial injury in podocytes of adriamycin-treated IRE1α KO mice. The difference in maximal OCR between adriamycin-treated control and IRE1α KO GECs was probably masked by the uncoupling of oxygen consumption and oxidative phosphorylation induced by FCCP. Mitochondrial OCR increased after TM treatment, in keeping with a previous report where ER stress was associated with mitochondrial calcium uptake and subsequent upregulation of OCR^9^. Decreased MitoTracker Red staining in resting IRE1α KO GECs correlates with reduced OCR. Since deletion of IRE1α did not affect mitochondrial biogenesis or mitochondrial mass, we propose that IRE1α modulates mitochondrial respiration at a functional level; for instance, through changes in mitochondrial membrane composition^47^, ER-mitochondrial calcium transfer^48^, or substrate import^49^.

In summary, IRE1α signaling is activated in experimental nephrosis. Importantly, the increased susceptibly to proteotoxic stress and glomerular disease caused by the deletion of IRE1α in podocytes is the result of a combined defect in multiple homeostasis processes. Beyond its function in the upregulation of chaperones and autophagy, IRE1α has a novel role in the regulation of mitochondrial bioenergetics. The relative contributions of ER chaperones, autophagy, and mitochondrial function to podocyte cytoprotection will require further study. Our findings establish IRE1α as an essential component of the podocyte proteostasis network and open the possibility of targeting the IRE1α pathway to improve proteostasis in chronic glomerular diseases, including human FSGS.

## Materials and methods

### Antibodies

Rabbit antibodies to IRE1α (3294), SQSTM1/p62 (5114), LC3B (2775), XBP1s (12782), cleaved-caspase-3 (Asp175) (9664), JNK (9252), phospho-JNK (Thr183/Tyr185) (4668), Beclin-1 (3495), and Bcl-2 (3498) were purchased from Cell Signaling Technology (Danvers, MA). Rabbit anti-Wilms tumor-1 (WT1; sc-192), goat anti-synaptopodin (sc-21537), and rat anti-GRP94 (sc-32249) were purchased from Santa Cruz Biotechnology (Santa Cruz, CA). Rabbit anti-GRP78/BiP and rabbit anti-ERp57 were from Enzo Life Sciences (Ann Arbor, MI). Rabbit anti-phospho-IRE1α (S724) (ab48187) was purchased from Abcam (Toronto, ON). Rabbit anti-actin (A2066) was from MilliporeSigma (Mississauga, ON). Goat anti-podocalyxin (AF1556) was purchased from R & D Systems (Minneapolis, MN). Rabbit anti-mesencephalic astrocyte-derived neurotrophic factor (MANF; ARMET; PAB13301) was purchased from Abnova (Walnut, CA). Rabbit anti-DNAJB11/ERdj3 (15484-1-AP) was from Proteintech (Rosemont, IL). Rabbit anti-peroxisome proliferator-activated receptor gamma coactivator 1-α (PGC1α; PA5-38021) was purchased from Thermo Fisher Scientific (Burlington, ON). Rabbit anti-nephrin antiserum was a gift from Dr. Tomoko Takano (McGill University)^50^. The horseradish peroxidase-conjugated antibodies goat anti-rabbit IgG (111-035-144), goat anti-rat IgG (112-035-003), and donkey anti-goat IgG (705-035-003) were purchased from Jackson ImmunoResearch Laboratories (West Grove, PA).

### Studies in mice

Generation, genotyping, and characterization of podocyte-specific IRE1α KO mice was described previously^22^. Briefly, mice with a floxed IRE1α gene (loxP sites surrounding exons 20 and 21) were bred with mice expressing Cre recombinase under control of the podocin promoter to obtain mice with podocyte-specific deletion of IRE1α, i.e. IRE1α^flox/flox^;Cre+ (IRE1α KO)^22^. The IRE1α construct results in an in-frame deletion of most of the ribonuclease domain, although a short peptide sequence identified by the anti-IRE1α antibody remains at the C-terminus. A shorter IRE1α ribonuclease-deleted protein is weakly detectable in cultured GECs (see below), but not in vivo (Supplementary Fig. 1A), indicating that the mutant protein is most likely unstable and degraded. Cre-mediated IRE1α gene deletion in glomeruli was demonstrated previously^22^. The expression of IRE1α protein is reduced in glomerular lysates of IRE1α KO mice, compared with littermate controls, i.e. IRE1α^flox/flox^;Cre- (Supplementary Fig. 1A, B). The IRE1α signal is not completely absent due to expression of IRE1α in glomerular mesangial and endothelial cells. Mice were housed in standard conditions with 12 h on-off light cycles and fed ad libitum. The animal protocol was approved by the McGill University Animal Care Committee. Studies were carried out in accordance with guidelines established by the Canadian Council on Animal Care.

Adult male IRE1α KO mice and control littermates (11–13 weeks old) received adriamycin (doxorubicin; MilliporeSigma) in a single injection through the tail vein at a dose that induces only modest injury in control mice (18 mg/kg)^45,50^. Untreated mice were also littermates. The number of mice required for this study was based on our previous experience with studies of adriamycin nephrosis^45,50^. Urine collections were performed weekly for 4 weeks until the mice were sacrificed. Urine albumin was quantified with an enzyme-linked immunosorbent assay (Mouse Albumin ELISA Quantification Kit, Bethyl Laboratories, Montgomery, TX). Albumin results were normalized to urine creatinine, which was measured using a picric acid-based reaction (Creatinine Colorimetric Assay Kit, Cayman Chemical Co.; Ann Arbor, MI).

For immunofluorescence microscopy, kidney sections were snap-frozen in HistoPrep (Fisher Scientific, Ottawa, ON) and stored at −80 °C immersed in 4-methylbutane. Tissues were sliced with a cryomicrotome (4 μm sections) and stained for podocalyxin or nephrin as described previously^22^. Images were captured in a Zeiss Axio Observer fluorescence microscope with visual output connected to an AxioCam MRm monochrome camera (Carl Zeiss AG; Toronto, ON). Quantification of immunofluorescence intensity was described previously^45,50^.

For light microscopy, kidney portions were fixed in 4% paraformaldehyde and stained with periodic acid-Schiff by conventional techniques at the McGill University Health Centre Histology Platform. Quantitative morphometry was used to characterize histological changes objectively (i.e. minimize observer bias). Slides were digitized at ×40 resolution in an Aperio AT Turbo scanner (Leica Biosystems, Buffalo Grove, IL). Images were processed using Aperio ImageScope 12.4 (Leica Biosystems). Glomeruli were randomly selected and analyzed with the Positive Pixel Count v9 algorithm, as reported previously^50^. Positive pixels were identified by a hue value of 0.854 (pink) and a hue width of 0.035^51^. Glomerular matrix expansion was expressed as the ratio of positive over total pixels (Supplementary Fig. 1C).

For transmission electron microscopy, kidney sections were fixed with 2.5% glutaraldehyde in cacodylate buffer (0.1 M sodium cacodylate, 0.1% calcium chloride, pH 7.4). Samples were imaged with a FEI Tecnai 12 electron microscope linked to an AMTV601 CCD camera at the McGill University Facility for Electron Microscopy Research. Quantitative analysis of electron micrographs was performed using Image J (National Institutes of Health, Bethesda, MD). Foot process width (FPW) was calculated with the formula: ${\mathrm{FPW}} = \frac{{{\uppi }} \times {\mathrm{GBM}}\,{\mathrm{lenght}}}{{4 \times {\mathrm{foot}}\,{\mathrm{process}}\,{\mathrm{number}}}}$ as described previously^52^. Mitochondrial circularity was determined by: ${\mathrm{Circularity}} = 4 \,\times \,{\uppi} \,\times\, \left( {\frac{{{\mathrm{Area}}}}{{{\mathrm{Perimeter}}^2}}} \right)$, where a circularity of 1 describes a perfect circle^53^. Mitochondrial matrix density was used as an additional parameter of mitochondrial health^54^, defined as: ${\mathrm{Density}} = \frac{1}{{{\mathrm{Mean}}\,{\mathrm{Pixel}}\,{\mathrm{Intensity}}}}$. Glomeruli were isolated by sequential sieving^22^.

### Studies in cell culture

Primary control and IRE1α KO cells were generated according to previously published methods^50^. Briefly, glomeruli were isolated from IRE1α^flox/flox^;Cre- mice and plated in type I collagen-coated plates to allow for outgrowth of primary GECs over the subsequent 6 days. Primary GECs were immortalized with a temperature-sensitive SV40 lentivirus^22^. Four clones were selected and expanded for further study, GECs were characterized by positive expression of nephrin, podocalyxin, synaptopodin, and WT1 proteins. Levels of these differentiation markers were evident at the proliferating temperature (33 ^o^C) and synaptopodin increased further when the cells were cultured at the differentiation temperature (37 ^o^C) for 6 days (Supplementary Fig. 2). A clone of GECs was transduced with a lentivirus encoding a tamoxifen-inducible Cre recombinase and mCherry fluorescent protein. After addition of tamoxifen, fluorescence-activated cell sorting was used to isolate highly fluorescent cells, where IRE1α had been edited through Cre–LoxP recombination (IRE1α KO). Immortalized GECs expressing full-length IRE1α served as controls.

GECs were incubated with tunicamycin (TM; 5 μg/mL) (MilliporeSigma) during 8 or 24 h to induce protein misfolding through inhibition of N-linked glycosylation. Dimethyl sulfoxide (BioShop; Burlington, ON) was the vehicle. Lysosomal acidification was inhibited with chloroquine (25 μM) (BioShop). The small molecule 4μ8C (20 μM) (Cayman Chemical Co.) was used to inhibit IRE1α RNA splicing^24^.

### Immunoblotting and immunoprecipitation

The protocol for immunoblotting was described previously^22,25,50^. Chemiluminescence signals were detected in a ChemiDoc Touch Imaging System (Bio-Rad; Mississauga, ON). Intensity of bands was quantified using ImageJ software, and the actin signal was used as loading control for normalization of signals. We ensured that the intensities of signals were within a linear range.

Coimmunoprecipitation of endogenous Bcl-2 and Beclin-1 was performed as described previously^29^. Cell lysates were precleared with agarose beads (MilliporeSigma). Samples were incubated with anti-Bcl-2 antibody overnight at 4 °C. Normal rabbit IgG (MilliporeSigma) was used as control. Protein–antibody complexes were pulled down by incubation with protein A-agarose beads (Abcam) for 2 h at 4 °C and eluted in SDS–PAGE sample buffer.

### Quantitative reverse transcriptase polymerase chain reaction

Total RNA was extracted with the RNeasy Mini Kit (Qiagen; Toronto, ON) following the manufacturer’s protocol. Genomic DNA removal and cDNA synthesis was done using the QuantiTect Reverse Transcription Kit (Qiagen), cDNA fragments were amplified with EvaGreen qPCR Mastermix (Applied Biological Materials; Richmond, BC) in a CFX384 Touch Real-Time PCR Detection System (Bio-Rad). Primer pairs are listed in Supplementary Table 1. Two reference genes (GAPDH and ACTB/β-Actin) were used to calculate relative expression^55^.

### Measurement of oxygen consumption rate

GECs were treated with TM (5 μg/mL) or adriamycin (1 μM) for 24 h. The cellular oxygen consumption rate (OCR) was measured in a Seahorse XFe96 extracellular flux analyzer (Agilent; Santa Clara, CA) using the Seahorse XF Cell Mito Stress Test Kit according to the manufacturer’s protocol^56^. The following mitochondrial inhibitors were used: oligomycin (1.5 µM), carbonyl cyanide 4-trifluoromethoxyphenylhydrazone (FCCP) (1.5 µM), rotenone (0.5 µM), and antimycin A (0.5 µM). OCR readings were adjusted to well absorbance^45^. The data were processed in Wave v2.6 (Agilent). ATP-linked OCR was calculated after inhibition of the ATP synthase with oligomycin, and maximal OCR was calculated after uncoupling oxygen consumption and ATP synthesis with FCCP.

### Measurement of mitochondrial mass

GECs were incubated for 30 min at 37 °C with 25 nM MitoTracker Green FM or 25 nM MitoTracker Red CMXRos (Thermo Fisher Scientific, Burlington, ON). Cells were washed, trypsinized, and analyzed in a LSRFortessa flow cytometer (BD Biosciences, San Jose, CA) as described^57^. The acquired data were analyzed using FlowJo v9 (BD Biosciences). Results were reported as mean fluorescence intensity of 20,000 cells, and values were normalized to the highest reading of each experiment.

GECs were plated on coverslips and labeled with MitoTracker Red CMXRos. Cells were fixed, permeabilized, and incubated with 1 µg/mL of fluorescein-phalloidin (Thermo Fisher Scientific) to stain the F-actin cytoskeleton. Images were captured at ×63 magnification in a fluorescence microscope. To quantify mitochondrial area, the mitochondrial network was selected through binary image thresholding in ImageJ^58^. Mitochondrial area was normalized to the cytoskeletal area.

### Nephroseq dataset analysis

The publicly accessible Nephroseq dataset “JuCKD” was used for the expression analysis of glomerular ER genes^59,60^. Nephroseq contains microarray gene expression data of laser-captured glomeruli from human kidney biopsies. The ER gene query was created by combining genes listed in the Protein Processing in the ER KEGG pathway (which includes UPR and other ER-related genes)^61^, and in the Qiagen human unfolded protein response PCR Array (PAHS-089Z, Qiagen). Of a total of 203 ER genes, 176 were present in the Nephroseq microarray. Principal component analysis of ER gene expression (algorithm that identifies the maximal variations in the data and reduces the dimensionality to a few components) was employed^60,62^. Pathway overrepresentation and gene ontology enrichment analysis were performed using the ConsensusPathDB interaction database^63^. Clinical characteristics and biochemical parameters of the patients were published previously^60^.

### Statistical analysis

All quantifications are reported as mean ± standard error. Data were processed in Prism 8.4 (GraphPad Software, La Jolla, CA). Comparisons between two groups were done by a two-tailed Student’s *t*-test. For three or more groups, statistical differences were assessed using one-way analysis of variance. When there were three or more groups and there were multiple biological replicates per animal/experiment in each group, two-way analysis of variance was used. Where significant differences were found, post-hoc analyses were performed using the Fisher’s exact test with Bonferroni correction. Significance scores are labeled as: * for *p* ≤ 0.05, ** for *p* ≤ 0.01, and *** for *p* ≤ 0.001. In studies of gene expression analysis, statistical significance was established using the Benjamini–Hochberg method.

## Supplementary information

Supplementary figure legends

Supplementary table legends

Supplementary Figure 1

Supplementary Figure 2

Supplementary Figure 3

Supplementary Figure 4

Supplementary Figure 5

Supplementary Figure 6

Supplementary Figure 7

Supplementary Figure 8

Supplementary Table 1

Supplementary Table 2

Supplementary Table 3

## References

[1] Wang M, Kaufman RJ (2016). Protein misfolding in the endoplasmic reticulum as a conduit to human disease. Nature.

[2] Hetz C, Papa FR (2018). The unfolded protein response and cell fate control. Mol. Cell.

[3] Karagoz, G. E., Acosta-Alvear, D. & Walter, P. The unfolded protein response: detecting and responding to fluctuations in the protein-folding capacity of the endoplasmic reticulum. *Cold Spring Harb. Perspect. Biol*. **11**, a033886 (2019). 10.1101/cshperspect.a033886 PMC6719602 30670466

[4] Rashid HO, Yadav RK, Kim HR, Chae HJ (2015). ER stress: autophagy induction, inhibition and selection. Autophagy.

[5] Klionsky DJ (2016). Guidelines for the use and interpretation of assays for monitoring autophagy (3rd edition). Autophagy.

[6] Margariti A (2013). XBP1 mRNA splicing triggers an autophagic response in endothelial cells through BECLIN-1 transcriptional activation. J. Biol. Chem..

[7] Sharma M (2017). Japanese encephalitis virus activates autophagy through XBP1 and ATF6 ER stress sensors in neuronal cells. J. Gen. Virol..

[8] Csordas G, Weaver D, Hajnoczky G (2018). Endoplasmic reticulum–mitochondrial contactology: structure and signaling functions. Trends Cell Biol..

[9] Bravo R (2011). Increased ER–mitochondrial coupling promotes mitochondrial respiration and bioenergetics during early phases of ER stress. J. Cell Sci..

[10] Ngoh GA, Papanicolaou KN, Walsh K (2012). Loss of mitofusin 2 promotes endoplasmic reticulum stress. J. Biol. Chem..

[11] Janikiewicz J (2018). Mitochondria-associated membranes in aging and senescence: structure, function, and dynamics. Cell Death Dis..

[12] Mori T, Hayashi T, Hayashi E, Su TP (2013). Sigma-1 receptor chaperone at the ER-mitochondrion interface mediates the mitochondrion–ER–nucleus signaling for cellular survival. PLoS ONE.

[13] Greka A, Mundel P (2012). Cell biology and pathology of podocytes. Annu. Rev. Physiol..

[14] Cybulsky AV (2017). Endoplasmic reticulum stress, the unfolded protein response and autophagy in kidney diseases. Nat. Rev. Nephrol..

[15] Cybulsky AV (2013). The intersecting roles of endoplasmic reticulum stress, ubiquitin- proteasome system, and autophagy in the pathogenesis of proteinuric kidney disease. Kidney Int..

[16] Tao J (2016). Endoplasmic reticulum stress predicts clinical response to cyclosporine treatment in primary membranous nephropathy. Am. J. Nephrol..

[17] Markan S (2009). Up regulation of the GRP-78 and GADD-153 and down regulation of Bcl-2 proteins in primary glomerular diseases: a possible involvement of the ER stress pathway in glomerulonephritis. Mol. Cell. Biochem..

[18] Madhusudhan T (2015). Defective podocyte insulin signalling through p85-XBP1 promotes ATF6-dependent maladaptive ER-stress response in diabetic nephropathy. Nat. Commun..

[19] Kawakami T (2015). Deficient autophagy results in mitochondrial dysfunction and FSGS. J. Am. Soc. Nephrol..

[20] Hartleben B (2010). Autophagy influences glomerular disease susceptibility and maintains podocyte homeostasis in aging mice. J. Clin. Investig..

[21] Cheng YC, Chang JM, Chen CA, Chen HC (2015). Autophagy modulates endoplasmic reticulum stress-induced cell death in podocytes: a protective role. Exp. Biol. Med..

[22] Kaufman DR, Papillon J, Larose L, Iwawaki T, Cybulsky AV (2017). Deletion of inositol-requiring enzyme-1alpha in podocytes disrupts glomerular capillary integrity and autophagy. Mol. Biol. Cell.

[23] Ali MM (2011). Structure of the Ire1 autophosphorylation complex and implications for the unfolded protein response. EMBO J..

[24] Cross BC (2012). The molecular basis for selective inhibition of unconventional mRNA splicing by an IRE1-binding small molecule. Proc. Natl Acad. Sci. USA.

[25] Tousson-Abouelazm, N., Papillon, J., Guillemette, J. & Cybulsky, A. V. Urinary ERdj3 and mesencephalic astrocyte-derived neutrophic factor identify endoplasmic reticulum stress in glomerular disease. *Lab. Investig.* **100**, 945–958 (2020). 10.1038/s41374-020-0416-5 32203149

[26] Shoulders MD (2013). Stress-independent activation of XBP1s and/or ATF6 reveals three functionally diverse ER proteostasis environments. Cell Rep..

[27] Lee AH, Iwakoshi NN, Glimcher LH (2003). XBP-1 regulates a subset of endoplasmic reticulum resident chaperone genes in the unfolded protein response. Mol. Cell. Biol..

[28] Yevshin I, Sharipov R, Kolmykov S, Kondrakhin Y, Kolpakov F (2019). GTRD: a database on gene transcription regulation-2019 update. Nucleic Acids Res..

[29] Fernandez AF (2018). Disruption of the beclin 1-BCL2 autophagy regulatory complex promotes longevity in mice. Nature.

[30] Pendergrass W, Wolf N, Poot M (2004). Efficacy of MitoTracker Green and CMXrosamine to measure changes in mitochondrial membrane potentials in living cells and tissues. Cytometry A.

[31] Ploumi C, Daskalaki I, Tavernarakis N (2017). Mitochondrial biogenesis and clearance: a balancing act. FEBS J..

[32] Yi M (2017). Autophagy is activated to protect against podocyte injury in adriamycin-induced nephropathy. Am. J. Physiol. Ren. Physiol..

[33] Di Malta C, Cinque L, Settembre C (2019). Transcriptional regulation of autophagy: mechanisms and diseases. Front. Cell Dev. Biol..

[34] Kishino A (2017). XBP1-FoxO1 interaction regulates ER stress-induced autophagy in auditory cells. Sci. Rep..

[35] Hetz C (2012). The unfolded protein response: controlling cell fate decisions under ER stress and beyond. Nat. Rev. Mol. Cell Biol..

[36] Maurel M, Chevet E, Tavernier J, Gerlo S (2014). Getting RIDD of RNA: IRE1 in cell fate regulation. Trends Biochem. Sci..

[37] Hetz, C., Zhang, K. & Kaufman, R. J. Mechanisms, regulation and functions of the unfolded protein response. *Nat. Rev. Mol. Cell Biol.* (2020). 10.1038/s41580-020-0250-z PMC8867924 32457508

[38] Raimundo N, Krisko A (2018). Cross-organelle communication at the core of longevity. Aging.

[39] Lee VW, Harris DC (2011). Adriamycin nephropathy: a model of focal segmental glomerulosclerosis. Nephrology.

[40] Chandran K (2009). Doxorubicin inactivates myocardial cytochrome c oxidase in rats: cardioprotection by Mito-Q. Biophys. J..

[41] Zhu C (2014). Dysfunction of the PGC-1alpha-mitochondria axis confers adriamycin-induced podocyte injury. Am. J. Physiol. Ren. Physiol..

[42] Arif E (2019). Mitochondrial biogenesis induced by the beta2-adrenergic receptor agonist formoterol accelerates podocyte recovery from glomerular injury. Kidney Int..

[43] Carreras-Sureda A (2019). Non-canonical function of IRE1alpha determines mitochondria-associated endoplasmic reticulum composition to control calcium transfer and bioenergetics. Nat. Cell Biol..

[44] Muller-Deile J, Schiffer M (2014). The podocyte power-plant disaster and its contribution to glomerulopathy. Front. Endocrinol..

[45] Elimam H, Papillon J, Guillemette J, Navarro-Betancourt JR, Cybulsky AV (2019). Genetic ablation of calcium-independent phospholipase A2gamma exacerbates glomerular injury in adriamycin nephrosis in mice. Sci. Rep..

[46] Casalena G (2014). Mpv17 in mitochondria protects podocytes against mitochondrial dysfunction and apoptosis in vivo and in vitro. Am. J. Physiol. Ren. Physiol..

[47] Colell A (2003). Cholesterol impairs the adenine nucleotide translocator-mediated mitochondrial permeability transition through altered membrane fluidity. J. Biol. Chem..

[48] De Stefani D, Rizzuto R, Pozzan T (2016). Enjoy the trip: calcium in mitochondria back and forth. Annu. Rev. Biochem..

[49] Rossi A (2020). Defective mitochondrial pyruvate flux affects cell bioenergetics in alzheimer’s disease-related models. Cell Rep..

[50] Woychyshyn B, Papillon J, Guillemette J, Navarro-Betancourt JR, Cybulsky AV (2020). Genetic ablation of SLK exacerbates glomerular injury in adriamycin nephrosis in mice. Am. J. Physiol. Ren. Physiol..

[51] Farris AB (2011). Morphometric and visual evaluation of fibrosis in renal biopsies. J. Am. Soc. Nephrol..

[52] van den Berg JG, van den Bergh Weerman MA, Assmann KJ, Weening JJ, Florquin S (2004). Podocyte foot process effacement is not correlated with the level of proteinuria in human glomerulopathies. Kidney Int..

[53] Kalkhoran, S. B. et al. Unique morphological characteristics of mitochondrial subtypes in the heart: the effect of ischemia and ischemic preconditioning. *Discoveries* **5**, (2017). 10.15190/d.2017.1 PMC5519153 28736742

[54] Gottlieb E, Armour SM, Harris MH, Thompson CB (2003). Mitochondrial membrane potential regulates matrix configuration and cytochrome c release during apoptosis. Cell Death Differ..

[55] Taylor SC (2019). The ultimate qPCR experiment: producing publication quality, reproducible data the first time. Trends Biotechnol..

[56] Nicholls DG (2010). Bioenergetic profile experiment using C2C12 myoblast cells. J. Vis. Exp.

[57] Puleston D (2015). Detection of mitochondrial mass, damage, and reactive oxygen species by flow cytometry. Cold Spring Harb. Protoc..

[58] Dagda RK (2009). Loss of PINK1 function promotes mitophagy through effects on oxidative stress and mitochondrial fission. J. Biol. Chem..

[59] Ju W (2013). Defining cell-type specificity at the transcriptional level in human disease. Genome Res..

[60] Chung CF (2019). Intrinsic tumor necrosis factor-alpha pathway is activated in a subset of patients with focal segmental glomerulosclerosis. PLoS ONE.

[61] Kanehisa M, Furumichi M, Tanabe M, Sato Y, Morishima K (2017). KEGG: new perspectives on genomes, pathways, diseases and drugs. Nucleic Acids Res..

[62] Ringner M (2008). What is principal component analysis?. Nat. Biotechnol..

[63] Kamburov A (2011). ConsensusPathDB: toward a more complete picture of cell biology. Nucleic Acids Res..

