# Supplementary material for: Role of IRE1α in podocyte proteostasis and mitochondrial health
Source: Cell Death Discov. 2020 Nov 19;6:128. doi: 10.1038/s41420-020-00361-4 (PMC7677398; doi:10.1038/s41420-020-00361-4)
Supplement: Supplementary file 1 — Supplementary figure legends [file 41420_2020_361_MOESM1_ESM.docx]

**Role of IRE1α in Podocyte Proteostasis and Mitochondrial Health**

José R. Navarro-Betancourt, Joan Papillon, Julie Guillemette, Takao Iwawaki, Chen-Fang Chung, and Andrey V. Cybulsky

**Supplementary Information**

Supplementary Figure Legends

**Supplementary Figure 1.** Characterization of IRE1α KO mice. (A) Representative immunoblot of IRE1α. The IRE1α flox/flox construct results in Cre-mediated in-frame deletion of most of the ribonuclease domain, although a short peptide sequence identified by the anti-IRE1α antibody remains at the C-terminus. Full-length IRE1α migrates at 130 kDa, and is evident in glomerular lysates of control (Ctrl) mice and control GECs in culture. TM upregulates expression of full-length IRE1α in control GECs. A shorter IRE1α ribonuclease-deleted protein is weakly detectable in cultured KO GECs, but not in vivo; expression of this protein is unaffected by TM. (A and B) Compared with control littermates, IRE1α levels are reduced significantly in glomeruli of podocyte-specific IRE1α KO mice. *** P≤ 0.001. (C) Glomerular matrix expansion was quantified with a pixel counting algorithm in Aperio Imagescope; pink areas were considered positive pixels. The pink area pixels in the PAS sections (C, left panel) were “marked up” as orange (C, right panel). Blue represents negative staining. Scale bar = 20 μM. (D and E) Expression of nephrin (NEPH) in glomerular lysates was not significantly different among groups (immunoblot and densitometric quantification).

**Supplementary Figure 2.** Characterization of cultured GECs. (A) Expression of the podocyte differentiation markers nephrin (NEPH), podocalyxin (PDX), synaptopodin (SYNPO), and WT1 in immortalized control (Ctrl) and IRE1α KO GECs was evident at the proliferation temperature (33^o^C). Levels of SYNPO increased significantly after culture at the differentiation temperature (37^o^C) for 6 days (representative immunoblots).

**Supplementary Figure 3.** Effect of IRE1α on the UPR in GECs. (A and B) Incubation with TM for 24 h stimulated secretion of the ER chaperones ERdj3 and MANF. Genetic deletion or chemical inhibition of IRE1α with 4μ8C reduced levels of MANF in media, but did not affect ERdj3 production (immunoblots). GRP94, an ER chaperone that is not secreted, is absent in the media and is shown for comparison. (C and D) After 8 h of TM treatment, expression of BiP, GRP94, and MANF increased in control (Ctrl) GECs. IRE1α deletion and 4μ8C treatment impaired the upregulation of GRP94, BiP, and MANF. **P≤ 0.01, *** P≤ 0.001. 3 experiments performed in duplicate.

**Supplementary Figure 4.** Effect of IRE1α on autophagy in GECs. (A and B) Compared with cells treated with chloroquine (CQ) alone, incubation with CQ and TM for 8 h did not induce LC3 lipidation (LC3-II; immunoblots). 3 experiments performed in duplicate.

**Supplementary Figure 5.** Effect of IRE1α on transcription of autophagy genes in GECs. (A) A publicly available gene transcription regulation database (GTRD) was used to identify autophagy-related genes that contain XBP1s binding sites and are potentially regulated by XBP1s. (B-D) Transcription of ATG12, BECN1 (Beclin-1), and PI3K catalytic subunit type 3 (PIK3C3; VPS34 in yeast) was upregulated after 24 h of TM treatment, but there were no differences between control (Ctrl) and IRE1α KO GECs (qPCR). *** P≤ 0.001. 3 experiments performed in duplicate.

**Supplementary Figure 6.** JNK activation and Beclin-Bcl2 interaction in GECs. (A and B) Activation-specific phosphorylation of JNK (Thr183/Tyr185) increased comparably in control (Ctrl) and IRE1α KO GECs after 24 h incubation with TM. For quantification, P-JNK was normalized to JNK. The change in GRP94 is shown for comparison (immunoblots). 2 experiments performed in duplicate. (C and D) Levels of Beclin-1 did not change with TM treatment. The change in IRE1α is shown for comparison. (E and F) Endogenous Bcl-2 and Beclin-1 were coimmunoprecipitated (IP) with an anti-Bcl-2 antibody. Immune complexes were immunoblotted with anti-Beclin-1 or anti-Bcl-2 antibodies. Stimulation with TM did not alter the Bcl-2-Beclin-1 interaction. 3 experiments performed in duplicate. WCL, whole cell lysate.

**Supplementary Figure 7.** Oxygen consumption rate (OCR), cell area, and expression of PGC1α. (A and B) Maximal and ATP-linked OCRs were determined with the Seahorse mitochondrial stress test; measurements from 3 experiments are expressed as the percentage of control. (A) Under resting conditions, IRE1α KO GECs have a lower maximal OCR than control GECs. (B) Deletion of IRE1α impairs ATP-dependent oxygen consumption under resting conditions and after adriamycin (ADR) injury. (C) Flow cytometry analysis. Forward scatter (FSC), which reflects cell size, is comparable in control (Ctrl) and IRE1α KO GECs. (D) GEC area was monitored by staining with fluorescein-phalloidin. IRE1α deletion or treatment with 4μ8C did not change cell area. 18-23 frames were measured per group in 3 experiments. (E and F) Control, 4μ8C-treated, and IRE1α KO GECs had similar levels of PGC1α under basal conditions and after 24 h of incubation with TM (representative immunoblot). 3 experiments performed in duplicate.

**Supplementary Figure 8.** Principal component analysis of changes in human glomerular ER gene expression. Upregulation of ER gene expression separates patients with FSGS from healthy controls.
