# Supplementary material for: Role of IRE1α in podocyte proteostasis and mitochondrial health
Source: Cell Death Discov. 2020 Nov 19;6:128. doi: 10.1038/s41420-020-00361-4 (PMC7677398; doi:10.1038/s41420-020-00361-4)
Supplement: Supplementary file 2 — Supplementary table legends [file 41420_2020_361_MOESM2_ESM.docx]

**Role of IRE1α in Podocyte Proteostasis and Mitochondrial Health**

José R. Navarro-Betancourt, Joan Papillon, Julie Guillemette, Takao Iwawaki, Chen-Fang Chung, and Andrey V. Cybulsky

**Supplementary Information**

Supplementary Table Legends

**Supplementary Table 1.** List of primers used for qPCR in mouse GECs.

**Supplementary Table 2.** Expression of ER-related genes in human FSGS. Fold change indicates expression in FSGS/Control. NS, not studied (ER genes not present in the Nephroseq microarray). Gene symbols in italics with blue highlight represent genes inducible by XBP1s.

**Supplementary Table 3.** Pathway overrepresentation and gene ontology enrichment analysis using all genes upregulated in FSGS patients. From a library of 11 933 genes, 2 064 were upregulated in FSGS patients. The upregulated genes are involved in processes that require UPR signaling.
