# Supplementary figures and images for: Role of IRE1α in podocyte proteostasis and mitochondrial health
Source: Cell Death Discov. 2020 Nov 19;6:128. doi: 10.1038/s41420-020-00361-4 (PMC7677398; doi:10.1038/s41420-020-00361-4)

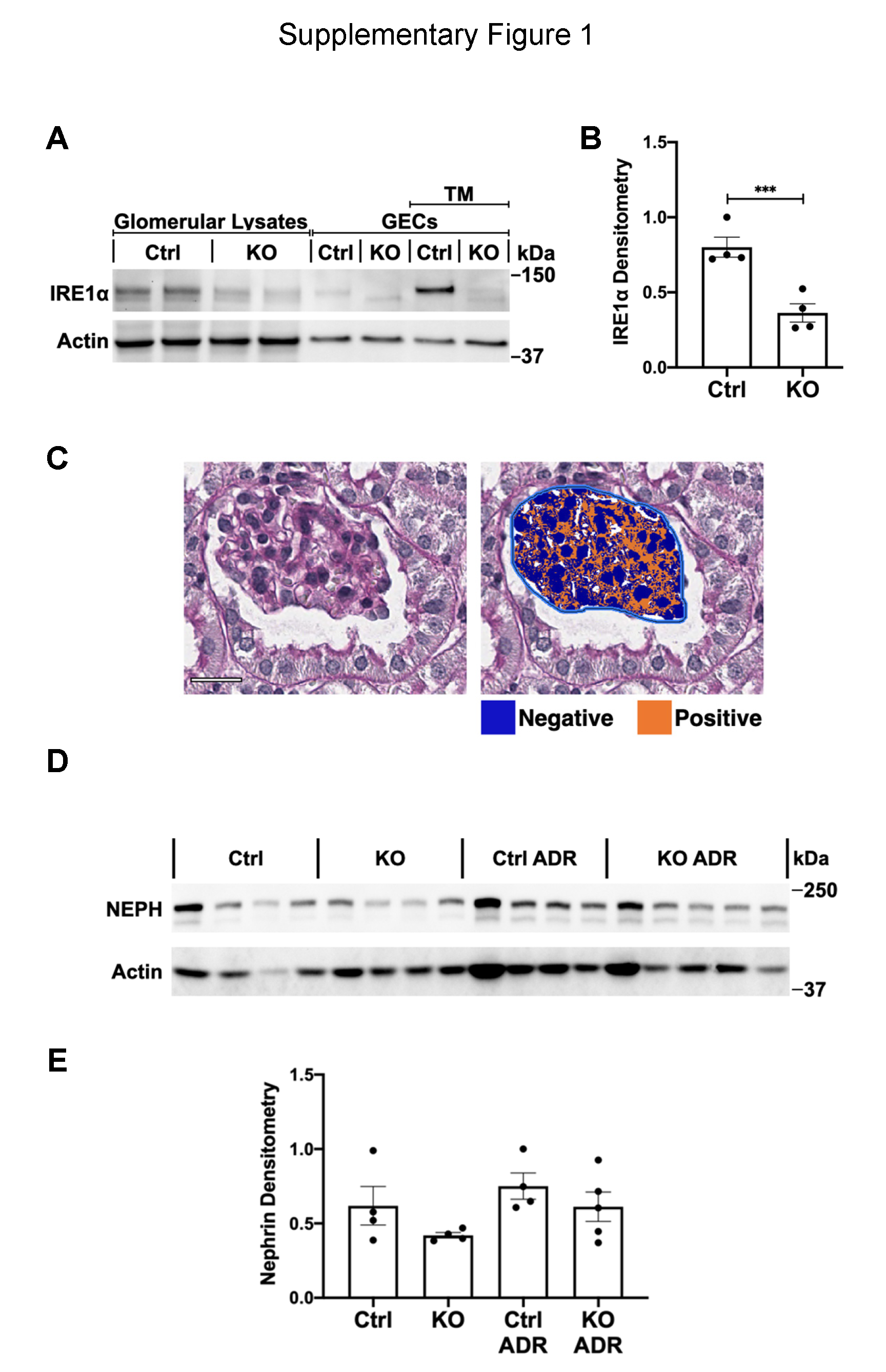

Supplement: Supplementary file 3 — Supplementary Figure 1 [file 41420_2020_361_MOESM3_ESM.tif]

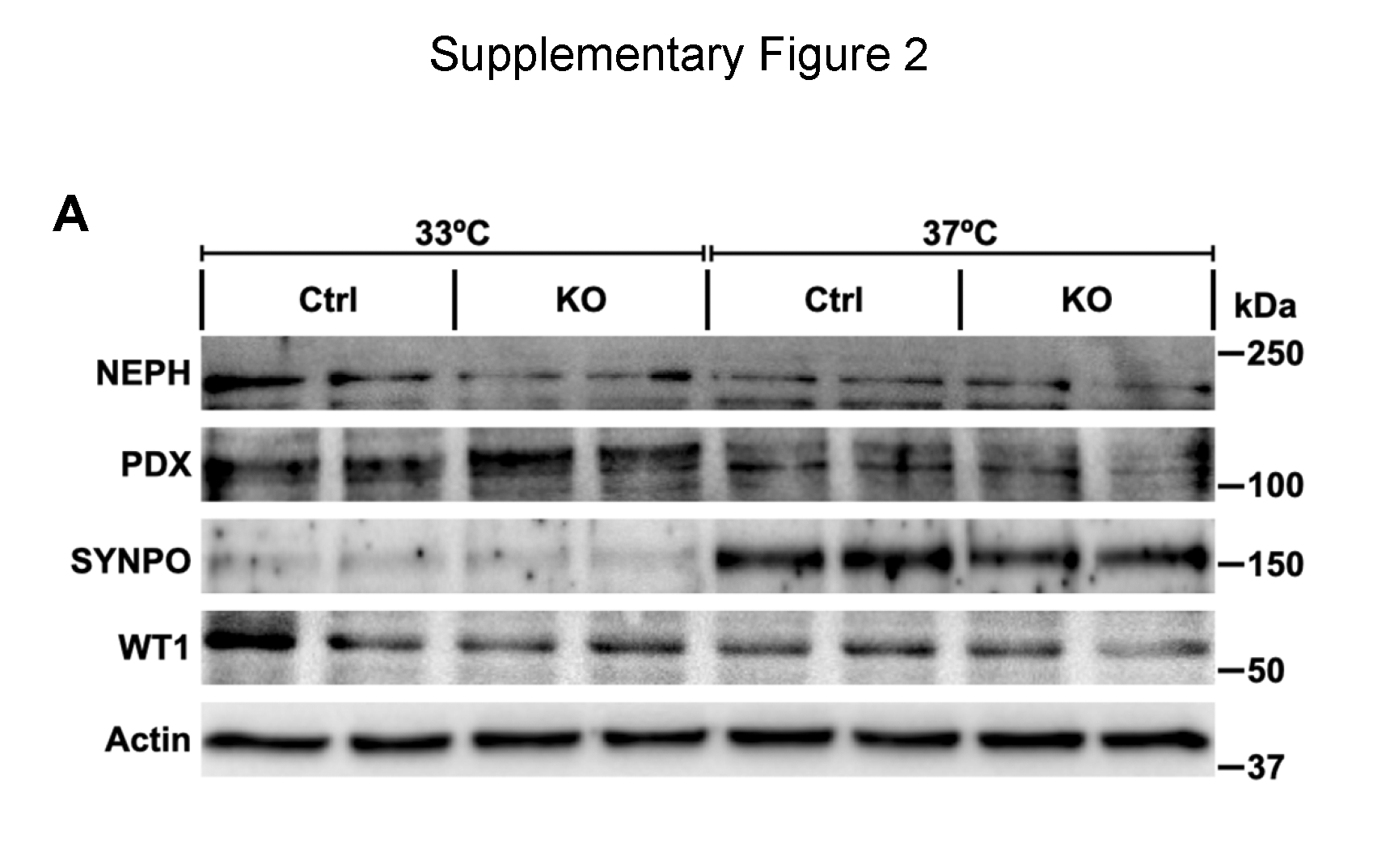

Supplement: Supplementary file 4 — Supplementary Figure 2 [file 41420_2020_361_MOESM4_ESM.tif]

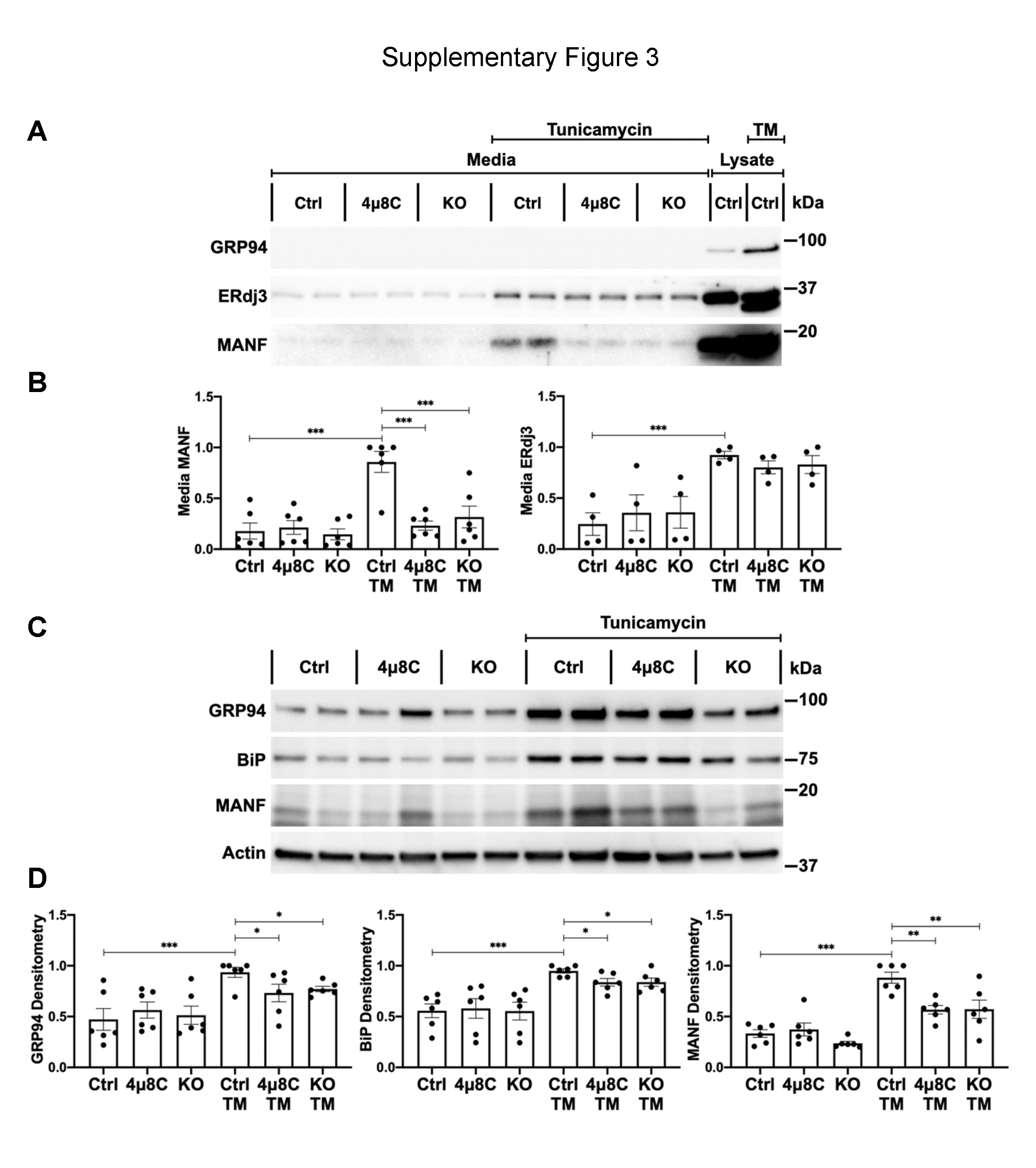

Supplement: Supplementary file 5 — Supplementary Figure 3 [file 41420_2020_361_MOESM5_ESM.tif]

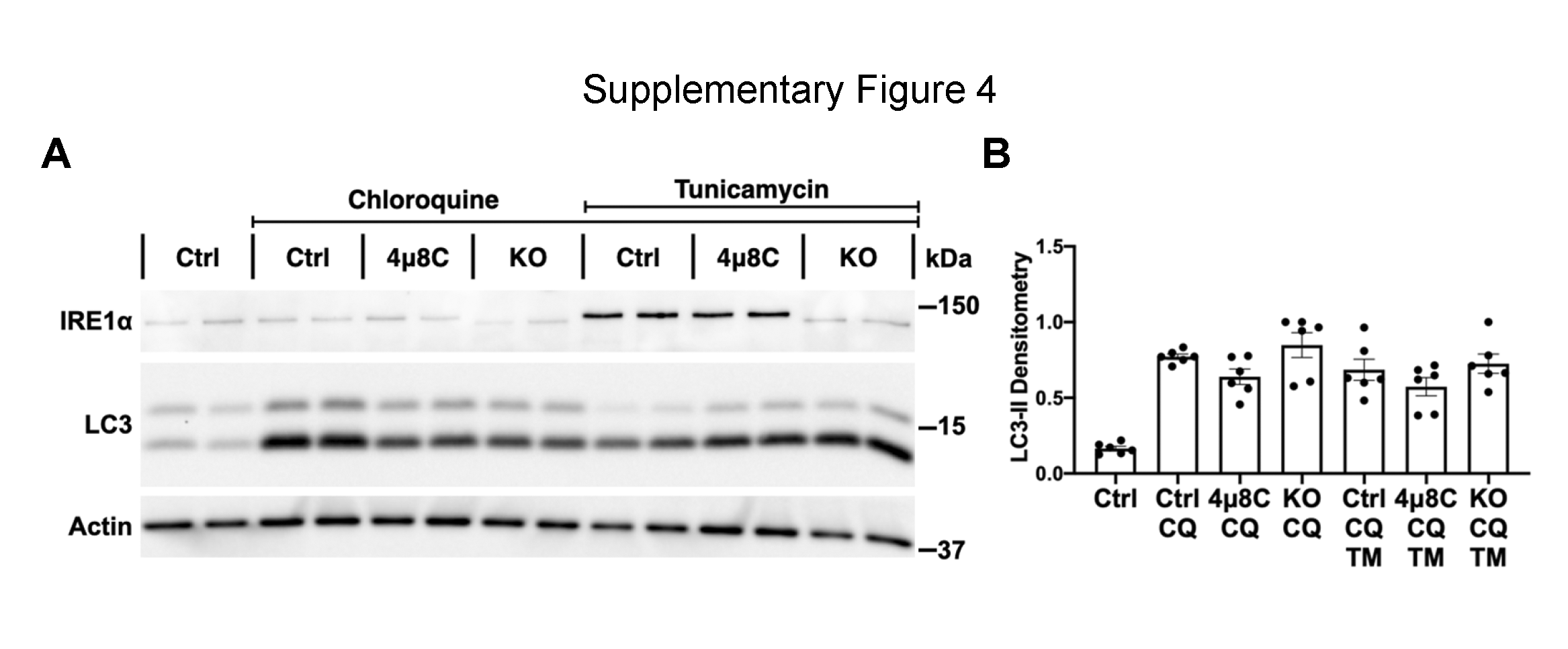

Supplement: Supplementary file 6 — Supplementary Figure 4 [file 41420_2020_361_MOESM6_ESM.tif]

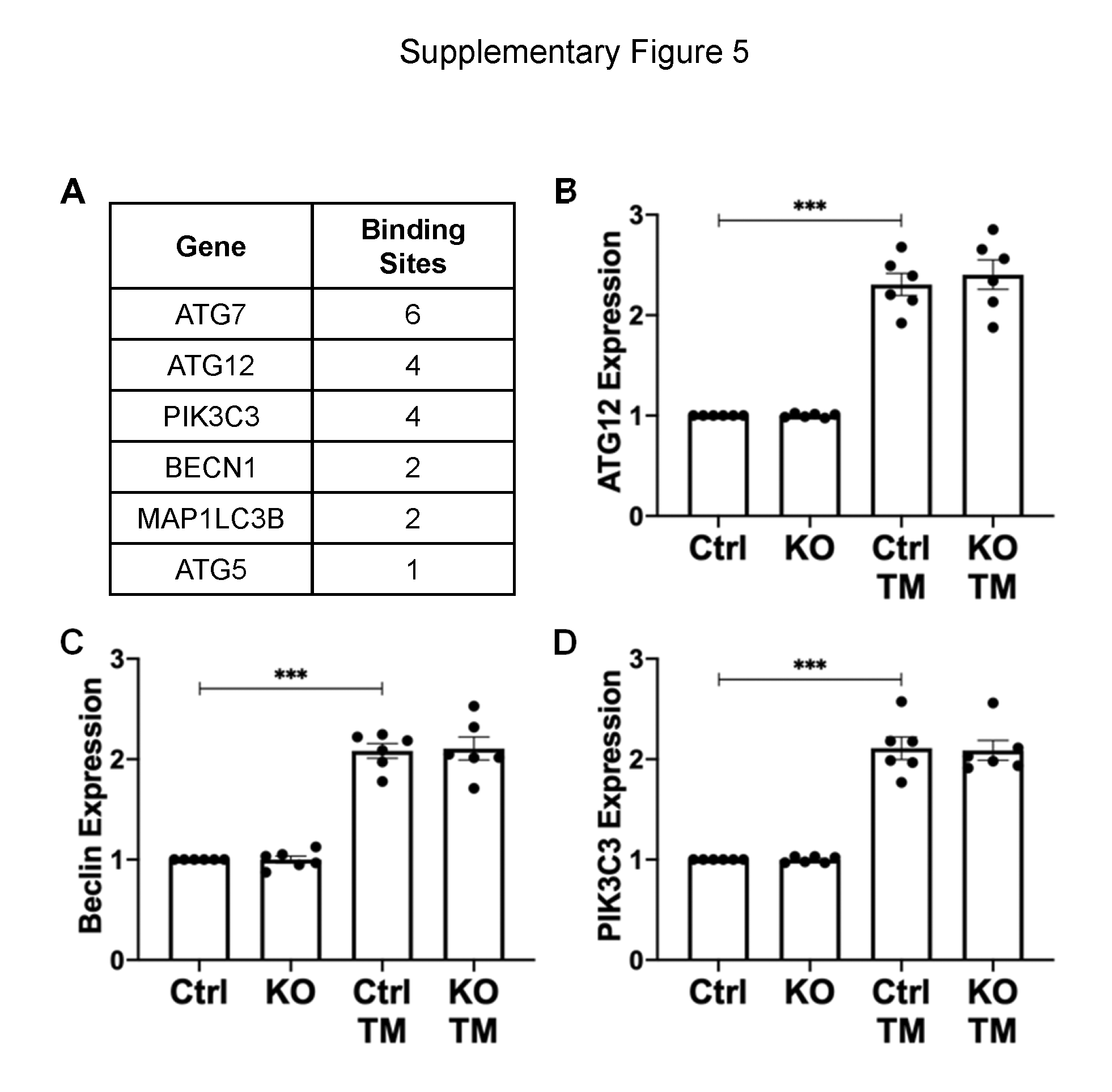

Supplement: Supplementary file 7 — Supplementary Figure 5 [file 41420_2020_361_MOESM7_ESM.tif]

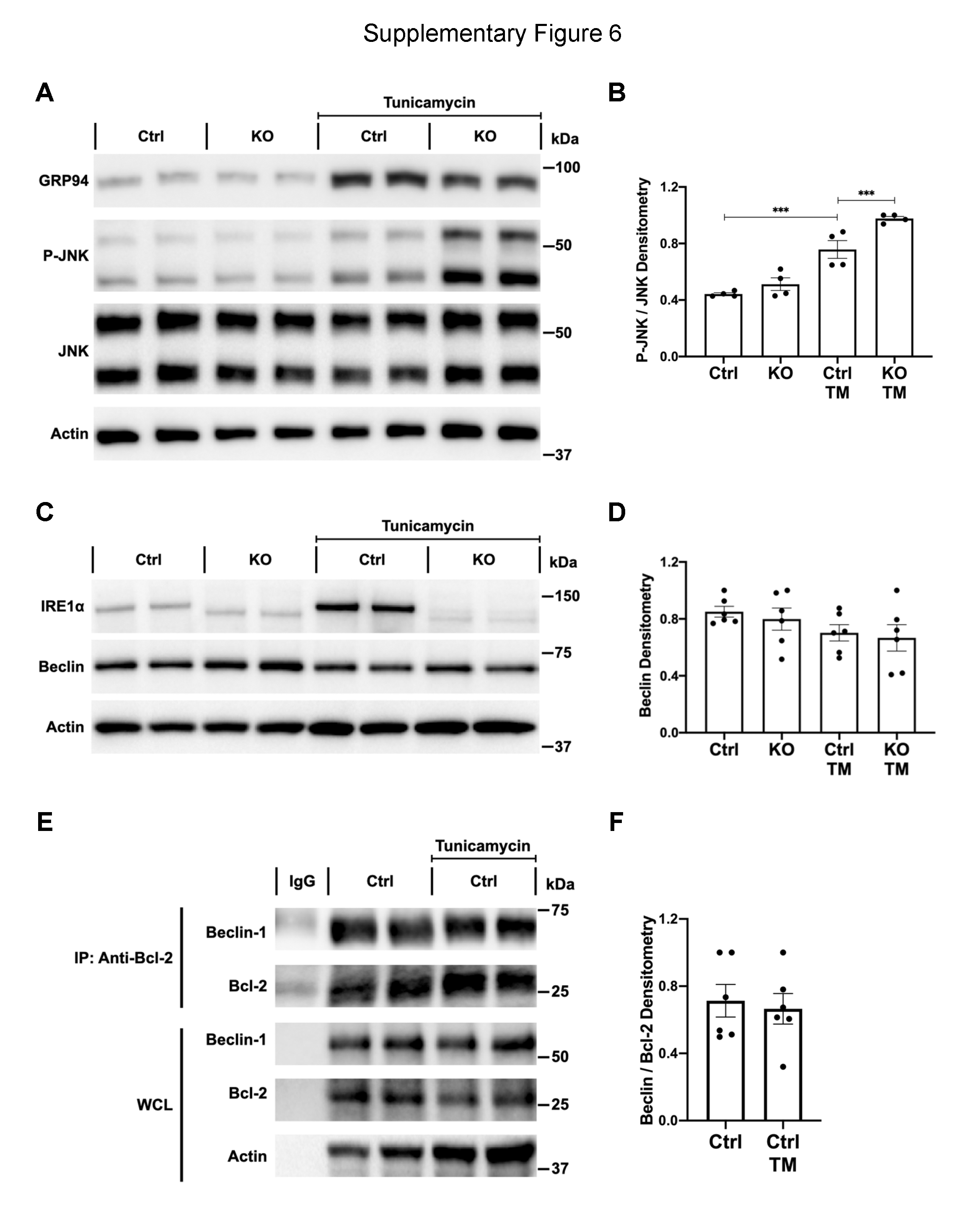

Supplement: Supplementary file 8 — Supplementary Figure 6 [file 41420_2020_361_MOESM8_ESM.tif]

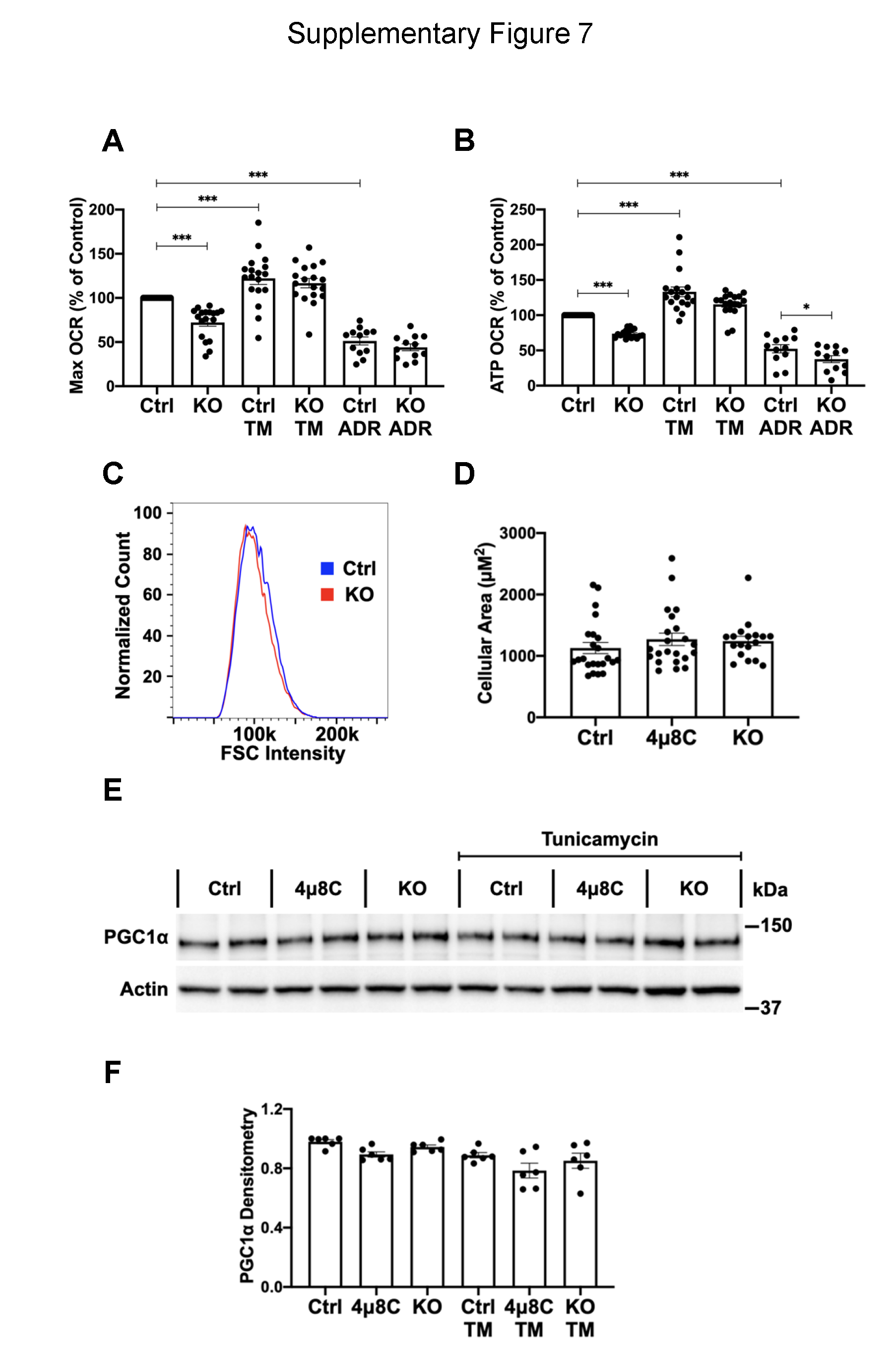

Supplement: Supplementary file 9 — Supplementary Figure 7 [file 41420_2020_361_MOESM9_ESM.tif]

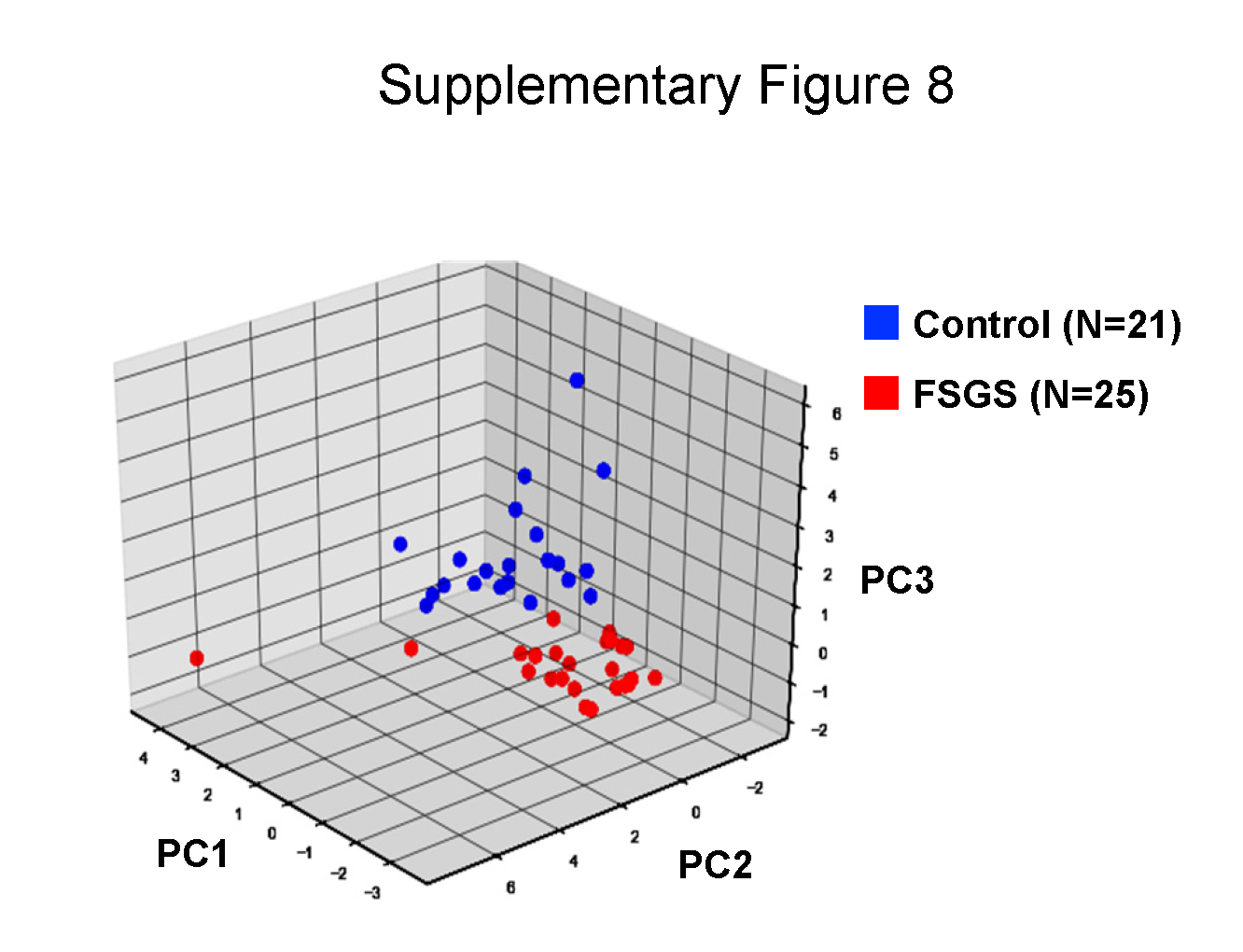

Supplement: Supplementary file 10 — Supplementary Figure 8 [file 41420_2020_361_MOESM10_ESM.tif]
